# Cell type-specific response of colon cancer tumor cell lines to oncolytic HSV-1 virotherapy in hypoxia

**DOI:** 10.1186/s12935-022-02564-4

**Published:** 2022-04-27

**Authors:** Sara Shayan, Arash Arashkia, Golnaz Bahramali, Asghar Abdoli, Mohammad Sadegh Shams Nosrati, Kayhan Azadmanesh

**Affiliations:** 1 Department of Molecular Virology, Pasteur Institute of Iran, Tehran, Iran; 2 Department of Hepatitis and AIDS and Blood Borne Diseases, Pasteur Institute of Iran, Tehran, Iran

**Keywords:** Oncolytic herpes virus type1, oHSV-1, HMGB1, CRC, Hypoxia, Normoxia

## Abstract

**Background:**

Novel strategies are required since the hypoxic tumor microenvironment is one of the important impediments for conventional cancer therapy. High mobility group box 1 (HMGB1) protein can block aerobic respiration in cancer cells. We hypothesized that HMGB1could also kill the colorectal cancer cells during hypoxia.

**Methods:**

In this study, we developed oncolytic herpes simplex virus type 1 expressing HMGB1 protein (HSV-HMGB1) and investigated the cytotoxic effect of HSV-HMGB1 and its parental virus (HSV-ble) on three colorectal cancer cells (HCT116, SW480, and HT29) under normoxic (20% oxygen) and hypoxic (1% oxygen) conditions. We further identified potential autophagy- related genes in HT29 cells by retrieving mRNA expression microarray datasets from the Gene Expression Omnibus database. These genes were then detected in HT29 cells infected with HSV-HMGB1 and HSV-ble during normoxia and hypoxia by Real-Time quantitative PCR (qRT-PCR).

**Results:**

The cytotoxic effect of HSV-HMGB1 was significantly higher than that of HSV-ble during normoxia; however, during hypoxia, HSV-HMGB1 enhanced the viability of HT29 cells at MOI 0.1. Analyzing the cell death pathway revealed that HSV-HMGB1 induced autophagy in HT29 cells under hypoxic conditions.

**Conclusion:**

In conclusion, it appears that oncolytic virotherapy is cell context-dependent. Therefore, understanding the cancer cells’ characteristics, microenvironment, and cell signaling are essential to improve the therapeutic strategies.

**Supplementary Information:**

The online version contains supplementary material available at 10.1186/s12935-022-02564-4.

## Importance

Colorectal cancer (CRC) is the third most common malignancy, and the hypoxic microenvironment plays a vital role in CRC pathogenesis. Innovative strategies seem to be highly required to overcome the hypoxic condition in CRC cells. Oncolytic virotherapy is one of the novel strategies for cancer therapy, and oncolytic herpes virus (oHSV-1, HHV-1 based on ICTV nomenclature) has been approved by FDA for melanoma treatment. However, cytotoxicity of oHSV against cancerous cells is reduced under hypoxic conditions. In this study, we designed an oHSV-1 to reduce the viability of CRC cells and explored the cell death mechanism during hypoxia and normoxia in vitro. The results of this study can provide an insight into the sensitivity of cancerous cells to recombinant oncolytic viral vectors under hypoxic conditions. Moreover, exploring cell death mechanisms may give future directions in manipulating viral vectors for being used alone or as a suitably-designed co-therapy.

## Introduction

The hypoxic microenvironment is commonly observed in solid tumors, including colorectal cancer (CRC). Shortage of oxygen results from inadequate oxygen delivery via inefficient tumor vasculature [1]. Tumor behavior is affected by hypoxia, and indeed, hypoxia can facilitate tumor progression and metastasis, leading to resistance to conventional chemo-radiotherapy [2]. Since CRC is the third most common malignancy and the fourth leading cause of cancer deaths worldwide [3], developing novel anticancer agents that efficiently kill tumor cells under hypoxic and normoxic conditions is required to improve clinical outcomes.

Oncolytic virotherapy can be regarded as a promising novel strategy for cancer therapy. Engineered oncolytic viruses are modified in a way that could selectively replicate in cancer cells without harming the normal tissues. Such viruses can be used as a delivery vehicle by harboring the gene of interest [4, 5]. Modified herpes simplex virus (HSV-1) has been investigated as an oncolytic virus candidate and has been FDA-approved for melanoma treatment recently. Other variants of the oncolytic HSV (oHSV-1) have also shown promising.

Results in some cancer diseases, including colon carcinoma [6]; however, this novel targeted treatment is not flawless. Friedman et al. showed replication, infectivity, and cytotoxicity of oHSV-1 was reduced under hypoxic conditions. Hence, oxygen tension should be regarded as an important variable when establishing next-generation oHSVs [7].

HMGB1 is a ubiquitous and conserved protein that is responsible for various biological activities inside and outside of the cells [8]. It has two nuclear localization signals (NLS1 and NLS2) and resides predominantly in the nucleus of eukaryotic cells while can rapidly be released into the cytosol. HMGB1 plays different roles in different redox states, and its biological function depends on its subcellular localization and expression. Additionally, its diverse isoforms play distinct roles in the pathogenesis of various diseases like colorectal cancers [9]. Gdynia et al. showed that HMGB1 could alter mitochondrial energy metabolism by blocking aerobic respiration, resulting in colorectal cancer cells death [10, 11]. It has also been shown that during hypoxia, nucleus-to-cytosol translocated HMGB1 binds to DNA, resulting in hepatocellular carcinoma proliferation [12]. However, in CRC and colorectal adenoma tissue samples, high rates of nuclear HMGB1 expression (84.0% and 92.6%, respectively) and moderate cytoplasmic HMGB1 expression (25.2% and 11.8%, respectively) have been reported [13]. Here, to determine whether oncolytic virotherapy and overexpression of HMGB1 under hypoxic conditions can affect subcellular localization of the protein and tumor cell death, we infected three colorectal cancer cell lines with a recombinant oHSV expressing HMGB1 and investigated its effects on the cell lines in in vitro normoxic and hypoxic environments.

## Results

### Construction and characterization of recombinant HSV-HMGB1

**Fig. 1:**
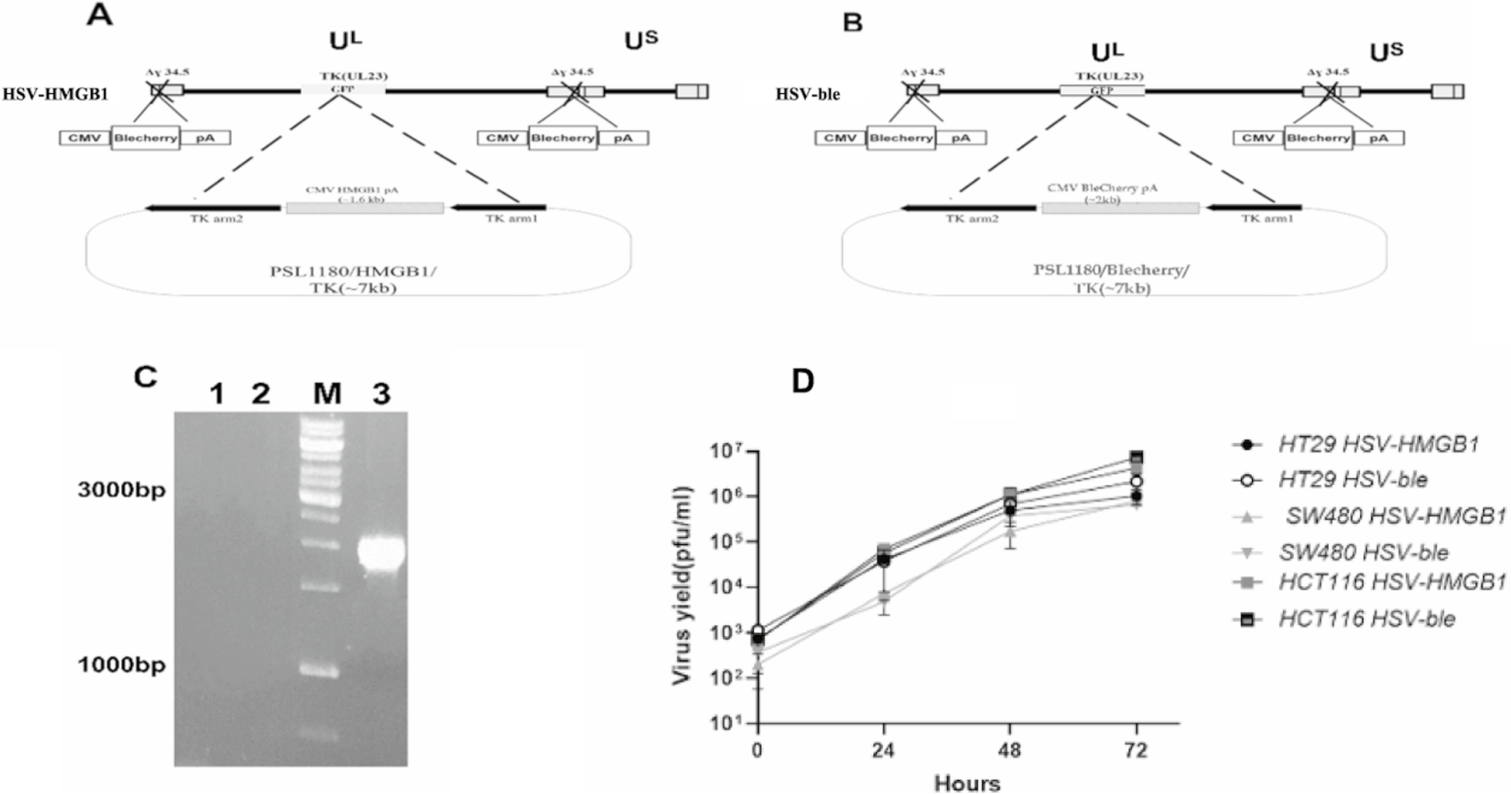
Construction and characterization of the HMGB1-harboring HSV. **A** and **B** Schematic diagram of herpes simplex virus showing the regions modified in HSV-HMGB1 and HSV-ble. The parental virus, HSV-ble, was generated by inserting Blecherry expression cassette into the tk locus. HSV/HMGB1 derived from HSV ICP34.5-Blecherry tk- GFP by inserting HMGB1 expression cassette into UL23 (TK) coding region. **C** PCR analysis of recombinant HSV-HMGB1. PCR was performed using DNA isolated from non-infected HEK293T, HSV-HMGB1 and HSV-ble by hmgb1F and tkF primers. Lane1: the PCR product of non-infected HEK293T; lane2: PCR product of HSV-ble; lane3: PCR product of HSV-HMGB1; Lane M: 1 kb DNA size marker. **D** Replication of HSV-HMGB1 and HSV-ble in human colorectal cancer cell lines. Cells were infected with HSV-HMGB1 and HSV-ble at MOI 0.1, and the virus yields were determined at 10, 24, 48 and 72 h post-infection

Construction of HSV ICP34.5- Blecherry was described in our previous study [14, 15]. The experiments were designed to construct recombinant viruses derived from HSV-1/Δ34.5/Blecherry in which both copies of the γ34.5 genes were disrupted and replaced by CMV-Blecherry-BGH poly-A cassette. The established system for the construction of the HSV-HMGB1 virus was composed of two steps. The first step required transfection of BHK cells with the shuttle vector, pSL1180-ARM1 and 2-HMGB1, a plasmid containing HMGB1 sequence under the control of CMV promoter flanked by tk homologous arms. In the second step, BHK cells were transfected with pSL1180-ARM1 and 2-HMGB1 and then infected with HSV-1/Δ34.5/Blecherry/tk-GFP to replace GFP with the HMGB1 expression cassette. By replacing the GFP sequence in HSV ICP34.5-Blecherry tk- GFP with Blecherry, HSV ICP34.5-Blecherry tk-BleCherry was constructed and used as a control; the recombinant red virus was obtained by harvesting the Blecherry positive plaques and isolating the virus by limiting dilution. Figure 1A and B depicts the recombination and genomic structure schema of HSV ICP34.5-Blecherry tk- BleCherry and HSV ICP34.5-Blecherry tk- HMGB1 hereafter called HSV-ble and HSV-HMGB1, respectively. In short, Vero cells were infected with HSV-HMGB1 at MOI 0.1, and recombinant red plaques were picked and subjected to plaque isolation to obtain the pure virus. The recombinant HSV-HMGB1 was further confirmed by PCR analysis and DNA sequencing of the *tk* locus. The 2000 bp PCR product represents the insertion of the targeted gene into *tk* deleted site (Fig. 1C). To assess the effect of HMGB1 on viral yield, replication of the two recombinant viruses was compared in HCT116, SW480, and HT29 cells, and then virus production was monitored over time using plaque assay. As shown in Fig. 1D, viral replication was similar for the two viruses. The replication of the new recombinant virus was not affected by carrying the transgene in the cells and conditions tested in this study.

### Basal expression of HMGB1 in colorectal cancer cell lines

**Fig. 2:**
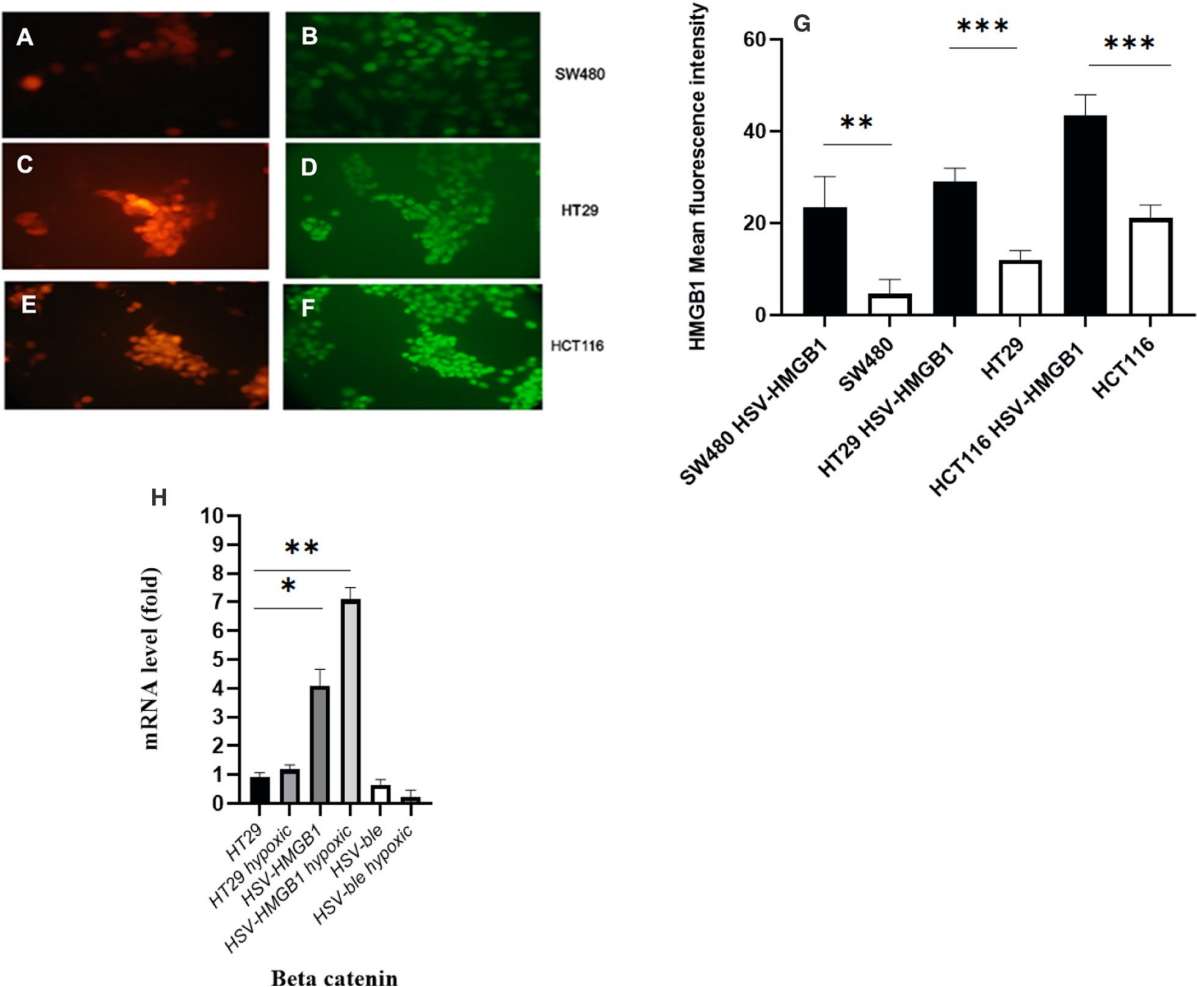
HMGB1 expression was significantly higher in CRC cell lines infected with HSV-HMGB1 than in uninfected cells. **A**, **C**, and **E** Infected CRC cells with HSV-HMGB1(red) at MOI 0.7. **B**, **D**, and **F** CRC cells stained with anti HMGB1 antibody (green). **B**, **D**, and **F** represent the same microscopic field of (**A**, **C**, and **E**). **G** Bar graphs show HMGB1 mean fluorescence intensity (MFI) in uninfected and HSV-HMGB1 infected cells. Fluorescence intensities were measured using the ImageJ software. **H** mRNA expression of Beta–catenin in HT29cells infected with HSV-HMGB1 and HSV-ble under hypoxic and normoxic condition were analysed by reverse transcription-quantitative PCR. Gene expression levels were calculated based on the Delta-Delta Ct relative quantification. Three biological replicates were performed *p < 0.05, **p < 0.01, and ***p < 0.001

An immunofluorescence experiment was used to determine the overexpression of HMGB1 in the infected colorectal cancer cell lines using anti HMGB1 antibody. Cells were infected with HSV-HMGB1 at MOI 0.7 (the dilution of the virus at which 50% of cells were infected), then the expression of HMGB1 was compared with uninfected cells in the same microscopic field, and Mean fluorescence intensities were quantified by ImageJ software. As shown in Fig. 2, (panel A, C and E indicates infected CRC cells with HSV-HMGB1(red) at MOI 0.7, and panel B, D and F indicates CRC cells stained with anti HMGB1 antibody (green)) based on fluorescence signal per cell, the mean basal expression of HMGB1 protein in uninfected HCT116 (mean ± SD, 21.25 ± 2.3 AU), SW480 (mean ± SD, 4.62 ± 1.21 AU) and HT29 cells (mean ± SD, 12 ± 1.95 AU) were significantly lower when compared to infected HCT116 (mean ± SD, 43.37 ± 2.1 AU), SW480 (mean ± SD, 23.5 ± 1.21 AU) and HT29 cells (mean ± SD, 29.1 ± 1.54 AU) (Fig. 2 panel G, and Table 1). To rule out fluorescence signal leaking from the Blecherry into the FITC channel, HT29 cells were infected with HSV-ble. Results showed no significant changes in HMGB1 expression between infected cells and uninfected cells. (Additional file 1: Fig. S1). Moreover, to confirm HMGB1 overexpression, Beta-catenin expression was evaluated in HT29 cells. Results indicate that Beta-catenin mRNA levels upregulated in HT29 cells infected with HSV-HMGB1 during normoxia and hypoxia (Fig. 2 panel H).

**Table 1:** Results of mean basal expression of HMGB1 protein in CRC cells

Infected and un-infected CRC cells	HMGB1 Mean Fluorescence Intensity
SW480 HSV-HMGB1	23.5
SW480	4.6
HT29 HSV-HMGB1	29.125
HT29	12
HCT116 HSV-HMGB1	43.37
HCT116	21.2

### Cytotoxicity activity of recombinant HSV-HMGB1

**Fig. 3:**
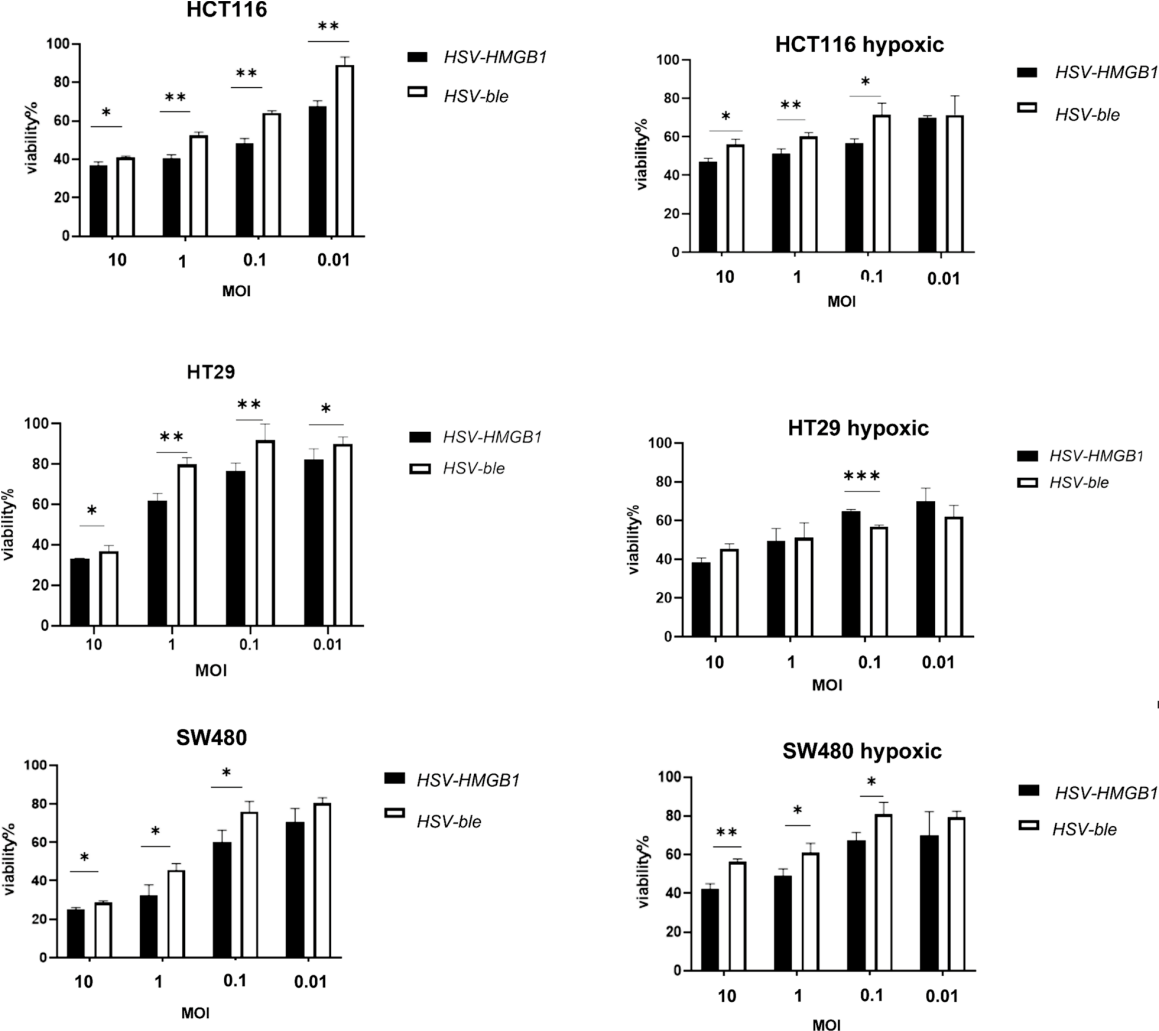
XTT assays with CRC cells under normoxic and hypoxic conditions demonstrating a dose-dependent cytotoxicity 72 h after infection with HSV-HMGB1, and HSV-ble. The relative cell viability was normalised to that of the control (non- infected CRC cells at 72 h). Three independent experiments were performed. For hypoxic conditioned HT29 cell five independent experiments were performed. data are shown as means ± sd. n = 3, *p < 0.05. **p < 0.01, and ***p < 0.001

The SW480, HCT116, and HT29 cells were infected with HSV-HMGB1 and HSV-ble at four different MOIs (0.01, 0.1, 1, and 10) under normoxic and hypoxic conditions. Seventy-two hours post-infection 2,3-bis-(2-methoxy-4-nitro-5-sulfophenyl)-2H-tetrazolium-5-carboxanilide (XTT) assay was used to determine the cytotoxicity of indicated viruses. As shown in Fig. 3, HSV-HMGB1 and HSV-ble could induce dose-dependent cancer cell-killing effect, and viability is more in case of hypoxia for both viruses at MOI 10 and 1 in HCT116 and SW480 cells (Additional file 1: Fig S2). In HT29 cells, however HSV-ble showed more cytotoxicity under hypoxic conditions. But cytotoxicity of HSV-HMGB1 in HT29 cells during normoxia and hypoxia was not statistically significant (Additional file 1: Fig. S2). HSV-HMGB1 elicited more efficient cytotoxic activity under hypoxic (*P* < 0.001 for HCT116, *P* < 0.05 for SW480) and normoxic (*P* < 0.001 for HCT116, *P* < 0.05 for SW480, and *P* < 0.001 for HT29) conditions than HSV-ble. But interestingly, in HT29 cells during hypoxia, HSV-ble showed superior tumor cell killing ability *P* < 0.01 than that of HSV-HMGB1. Of note, the experiment was repeated five times with similar results, but the result needs to be interpreted cautiously.

### Examining the apoptotic activity of the recombinant HSV-HMGB1

**Fig. 4:**
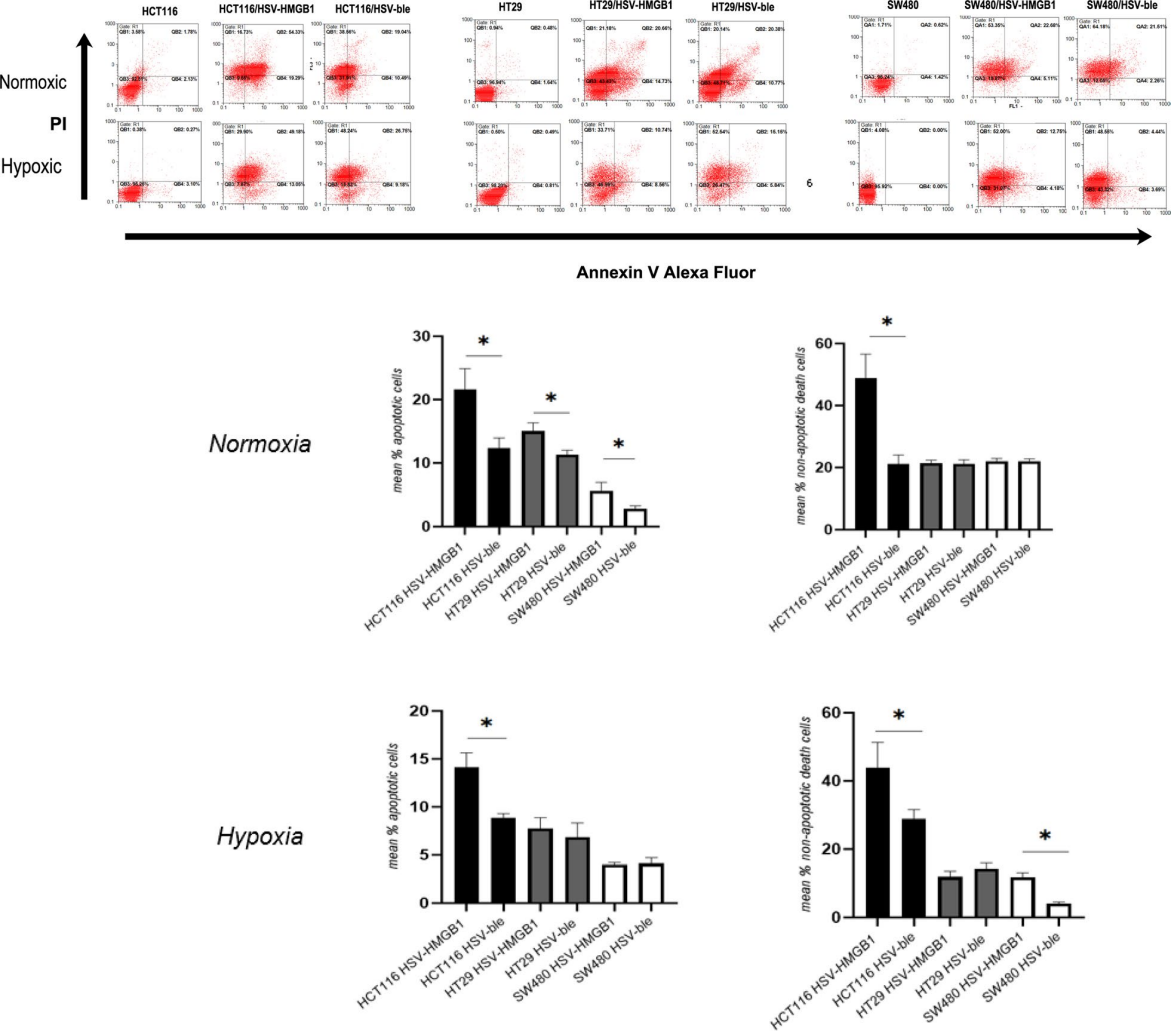
Apoptosis analysis by Annexin-V/PI double staining of HCT116, HT29, and SW480 cells infected with HSV-HMGB1 and HSV-ble at MOI 0.1 for 24 h under hypoxic and normoxic condition. Percentages of Annexin-V positive and Annexin-V/PI double-positive cells are shown in the QB4 and QB2 quadrants respectively. **B** Bar graph representing percentages of apoptotic cells (Annexin-V positive, PI negative) and non-apoptotic dead cells (Annexin-V positive, PI positive) as measured by flow cytometry. Data are shown as means ± s.d. At least two independent experiments were performed., *p < 0.05

Considering the influence of hypoxia on the cytotoxic activity of HSV-HMGB1, we wondered whether the recombinant virus could modulate apoptosis in the host cells [16, 17]. In fact, HMGB1 plays a key role in regulating apoptosis and autophagy [18]. Accordingly, Annexin-V-Alexa Flour and PI staining were performed to quantify cell apoptosis. As seen in Fig. 4, and Table 2, in HCT116 cells, Annexin/PI double staining revealed that the apoptosis following HSV-HMGB1 infection has increased to 54.3% and 49.1%, that were nearly 2.8 and 1.8 times more than that of HSV-ble 19% and 26.7% during normoxia and hypoxia, respectively. Under the normoxic conditions, early apoptosis (Annexin-V positive, PI negative) induced by HSV-HMGB1 was increased (14.7% for HT29 and 5.1% for SW480 cells) in comparison with HSV-ble (10.7% for HT29 and 2.2% for SW 480); and there were no notable changes in the percentage of the late-stage of apoptotic cells. Even though during hypoxia, the percentage of Annexin/PI-positive SW480 cells infected with HSV-HMGB1 decreased (12.7% compared to 22.6% in normoxic conditions), it is still three times more than apoptosis in SW480 cells infected with HSV-ble during hypoxia (4.8%). Interestingly, the rate of early and late apoptosis is about twice in the case of normoxic conditions (compared to hypoxic) for HSV-HMGB1-infected HT29. Our data showed no differences in the rate of early apoptosis in HT29 cells infected with HSV-HMGB1 and HSV-ble infected under hypoxic conditions. Of note, since the sensitivity and condition of the XTT assay and Annexin-PI assay are different, we assumed that we could not directly compare the two results; however, as can be seen in Figs. 3 and 4, the ability of viruses for killing the CRC cells followed the similar pattern in both assays.

**Table 2:** Results of Annexin V/PI staining for cell apoptosis after HSV-HMGB1 1, and HSV-ble infection under normoxic or hypoxic condition

CRC cells	Early Apoptotic (%)	Late Apoptotic/Necrotic (%)	Total Apoptotic (%)
HCT116 HSV-HMGB1	19.29	54.33	73.62
HCT116 HSV-ble	10.49	19.04	29.53
HCT116 HSV-HMGB1 Hypoxia	13.05	49.18	62.23
HCT116 HSV-ble Hypoxia	9.18	26.75	35.93
HT29 HSV-HMGB1	14.73	20.66	35.39
HT29 HSV-ble	10.77	20.38	31.15
HT29 HSV-HMGB1 Hypoxia	8.56	10.74	19.3
HT29 HSV-ble Hypoxia	5.84	15.15	20.99
SW480 HSV-HMGB1	5.11	22.68	27.79
SW480 HSV-ble	2.26	21.51	23.77
SW480 HSV-HMGB1 Hypoxia	4.18	12.75	16.93
SW480 HSV-ble Hypoxia	3.69	4.44	8.13

### Analyzing autophagy following infection with the recombinant HSV-HMGB1

**Fig. 5:**
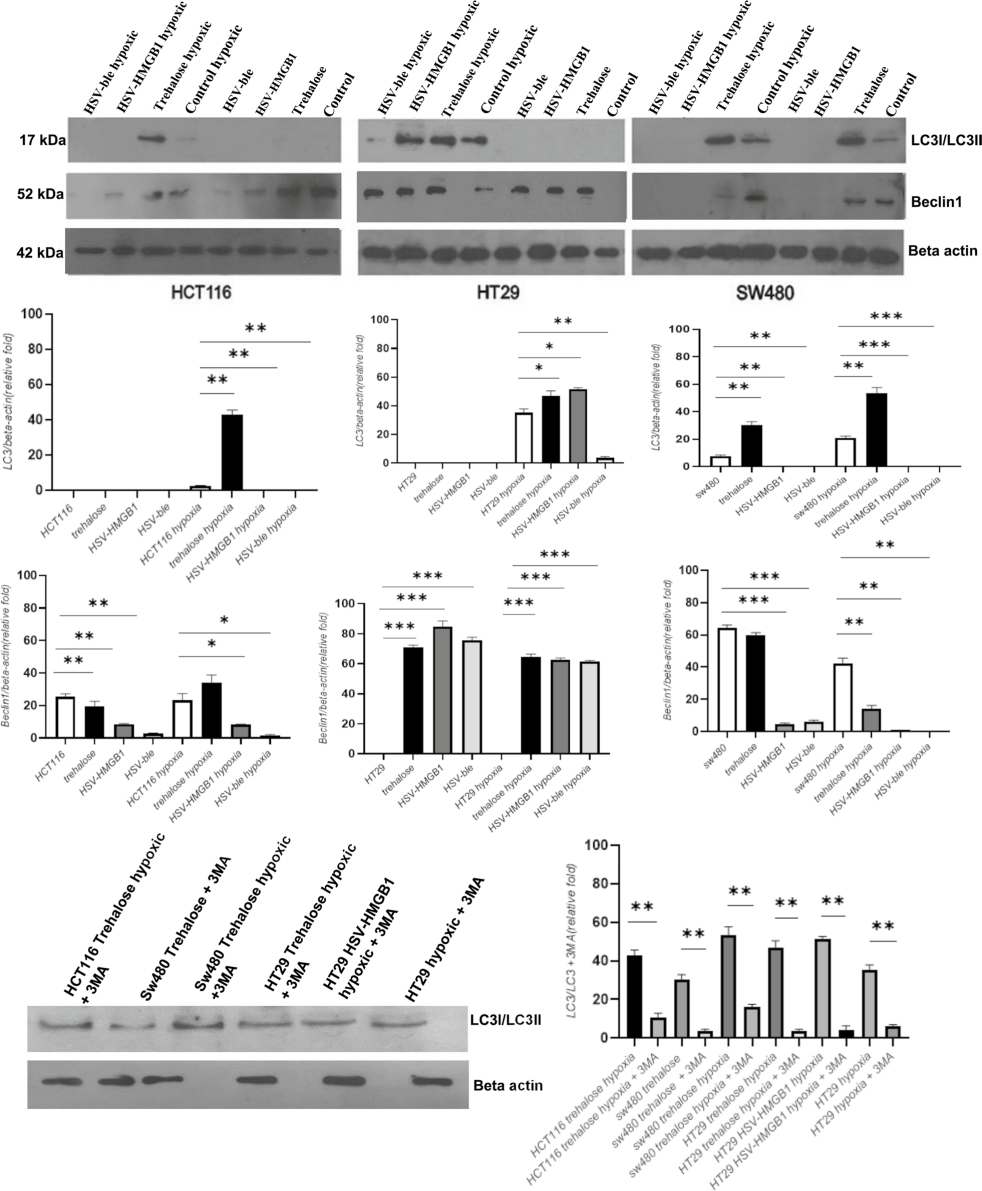
**A** Immunoblot analysis of HSV-HMGB1 and HSV-ble infected CRC cell lines. HCT116, HT29 and SW480 were infected with HSV-HMGB1 and HSV-ble at MOI 0.1 under hypoxic and normoxic conditions. The cell lines were also treated with 100 mM Trehalose for 24 h. Cells were harvested after 24 h and subjected to immunoblotting using anti-LC3, and anti-Beclin1 antibody. An antibody against β-actin was used to normalize blotted protein levels. Expression of LC3 and Beclin1was quantified using Image J software. The standard deviation was used to assess data dispersion. At least two independent experiments were performed. **B** LC3 positive cells were preincubated in the presence of 10 mM of 3-methyladenine (3MA) for 20 min then expression of LC3-II formation was investigated by western blotting with *β*-actin being an internal control. Expression of LC3 and LC3 + 3MA was quantified using Image J software. The standard deviation was used to assess data dispersion. At least two independent experiments were performed.*p < 0.05, **p < 0.01, and ***p < 0.001

Based on the apoptosis analysis, the higher viability of the HSV-HMGB1-infected HT29 cell line under hypoxic conditions may not be due to the apoptosis. So, further investigation was conducted by measuring the expression level of microtubule-associated protein light chain 3 (LC3) to monitor autophagy in the colorectal cancer cell lines [19]. To investigate whether autophagy activation was increased in HSV-HMGB1 infected cells, the cell lines were infected with HSV-HMGB1 or parental HSV-ble at MOI 0.1. The LC3-II expression was examined by immunoblotting 24 h after infection under hypoxic and normoxic conditions. Of note, Trehalose is widely considered as a potent autophagic inducer [20] and was used as a control to induce LC3-II expression in the three cell lines. The result in Fig. 5A indicated that LC3-II expression level was increased in all three cell lines during hypoxia. Trehalose induced the expression of LC3-II protein in HCT116, SW480, and HT29 under hypoxic conditions, but was not be able to increase LC3-II in HCT116 and HT29 cells during normoxia. Moreover, HSV-HMGB1 and HSV-ble failed to induce LC3-II under hypoxic conditions in all the three cell lines except for HT29 cells, in which HSV-HMGB1 caused an increase in LC3-II expression. Based on Fig. 5, it can be deduced that LC3 expression can vary among the three CRC cell lines in response to different stresses and, more importantly, the induction of LC3 might be counteracted by oncolytic herpes infection except for HSV-HMGB1 infected HT29 cells during hypoxia; our findings are aligned with the previous study by Lussingol et al. which identified HSV1 US11 protein could inhibit autophagy by direct interaction with PKR [21]. 3-Methyladenine (3-MA) has been used widely as an autophagy inhibitor; therefore, 3-MA was used to clarify the effect of autophagy in our study. As shown by western blot, 3-MA pretreatment significantly decreased the LC3II expression in LC3 positive CRC cells (Fig. 5B). We further examined the Beclin1 expression as it has a central role in autophagosome formation [22]. Our data revealed that Beclin1 expression significantly decreased in HCT116 and SW480 cells infected with HSV-HMGB1 and HSV-ble under hypoxic and normoxic conditions, whereas HSV-HMGB1 and HSV-ble infected HT29 cells showed no significant differences in the expression level of Beclin1 during hypoxia or normoxia. Overall, it can be assumed that HSV-HMGB1 induces autophagy only in HT29 cells during hypoxia.

### Subcellular localization of p53 and HMGB1 in HSV-HMGB1 infected colorectal cancer cell lines

**Fig. 6:**
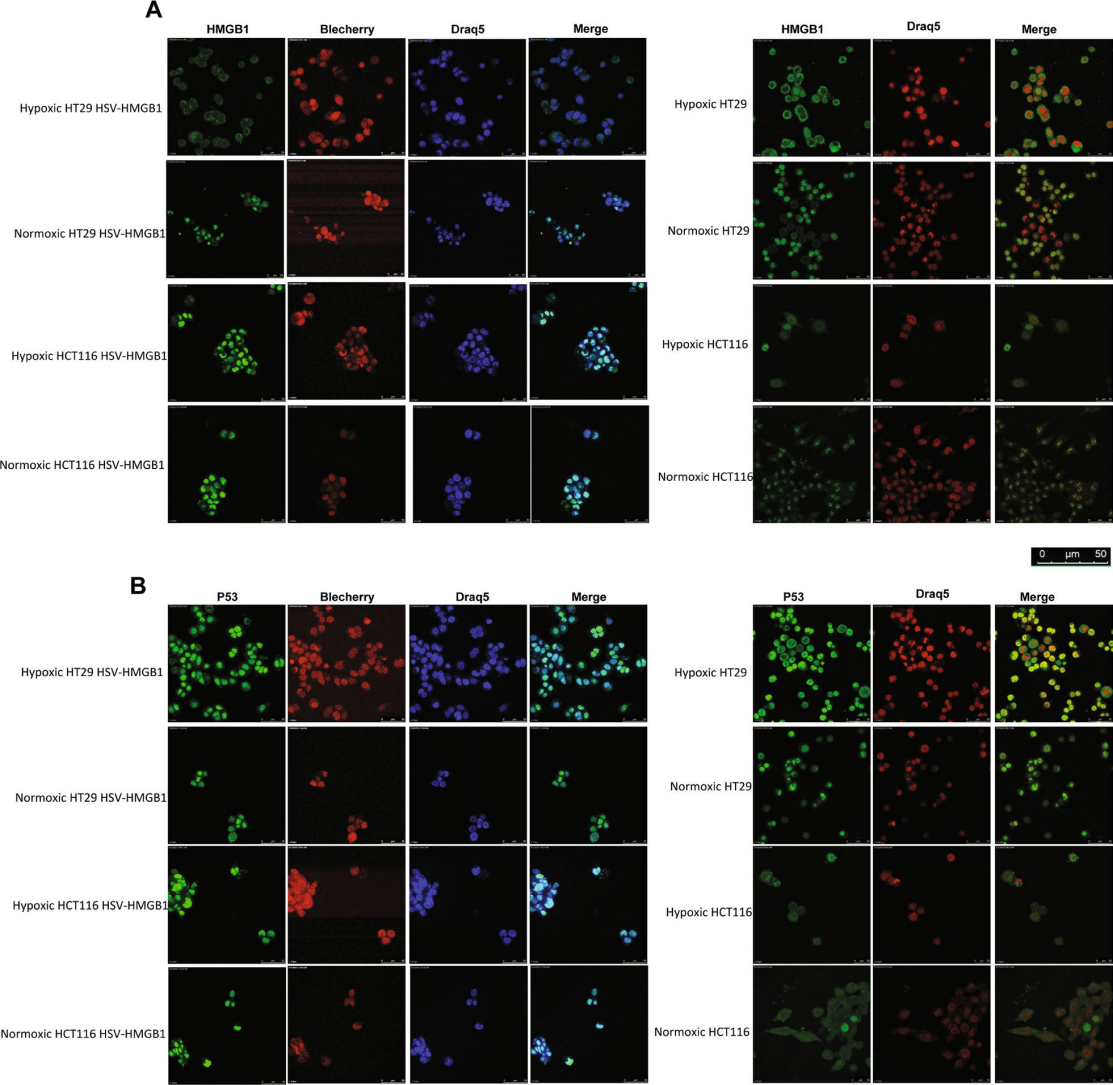
Confocal microscopy analysis of HMGB and p53 localization. 3 × 10^4^ Cells were fixed, permeabilized and stained for HMGB1 or p53 (green). Infected HCT116 and HT29 cells emitting red light. Draq5 was used to visualize the nuclei. **A** After 24 h, localization of HMGB1 and P53 in HT29 (mutated p53) and HCT116 (wild p53) infected with HSV-HMGB1 at MOI 0.1were analyzed under hypoxic and normoxic condition. **B** Localization of HMGB1 and P53 in uninfected HT29 and HCT116 cells were also analyzed during hypoxia and normoxia. All data are representative of two experiments. Scale bar: 50 µm

Several studies have shown that HMGB1/p53 complexes could regulate the cell death pathway [23]. Hence, to determine if an increased level of autophagy in hypoxic HT29 cells infected with HSV-HMGB1, as demonstrated by increased LC3-II expression, is correlated with localization of p53 and HMGB1, we analyzed the subcellular localization of HMGB1 and p53 in mock and HCT116 (harboring wild type p53) and HT29 (containing mutated p53) cells infected with HSV-HMGB1 under hypoxic and normoxic conditions (Fig. 6A and B) [24]. Of note, Since HSV-HMGB1 induces autophagy in HT29 cells under hypoxic conditions, we explored the localization of HMGB1 and P53 in HSV-HMGB1 in HT29 cells (HT29 cells contain mutated p53), and we used HCT116 cells, which contain the wild type of p53 as a control cell. Interestingly, confocal microscopy revealed that during hypoxia, HMGB1 protein predominantly translocated to cytoplasm while the majority of p53 resides in the nucleus of HT29 cells infected with HSV-HMGB1 (Additional file 1: Fig. S3); a similar image was also observed with hypoxic conditioned HT29 cells (not-virus treated) (Fig. 6A). However, during normoxia, p53 and HMGB1 were both accumulated in the nucleus of HT29 cells infected with HSV-HMGB1 (Fig. 6B). Furthermore, the level of cytosolic p53 was higher in HCT116 than in HCT116 cells infected with HSV-HMGB1 during hypoxia (Additional file 1: Fig. S3). These findings indicated that HMGB1 and p53 interactions in the nucleus might help to limit autophagy, and cytoplasmic HMGB1 might be responsible for autophagy activation.

### Identification of differentially expressed genes

**Fig. 7:**
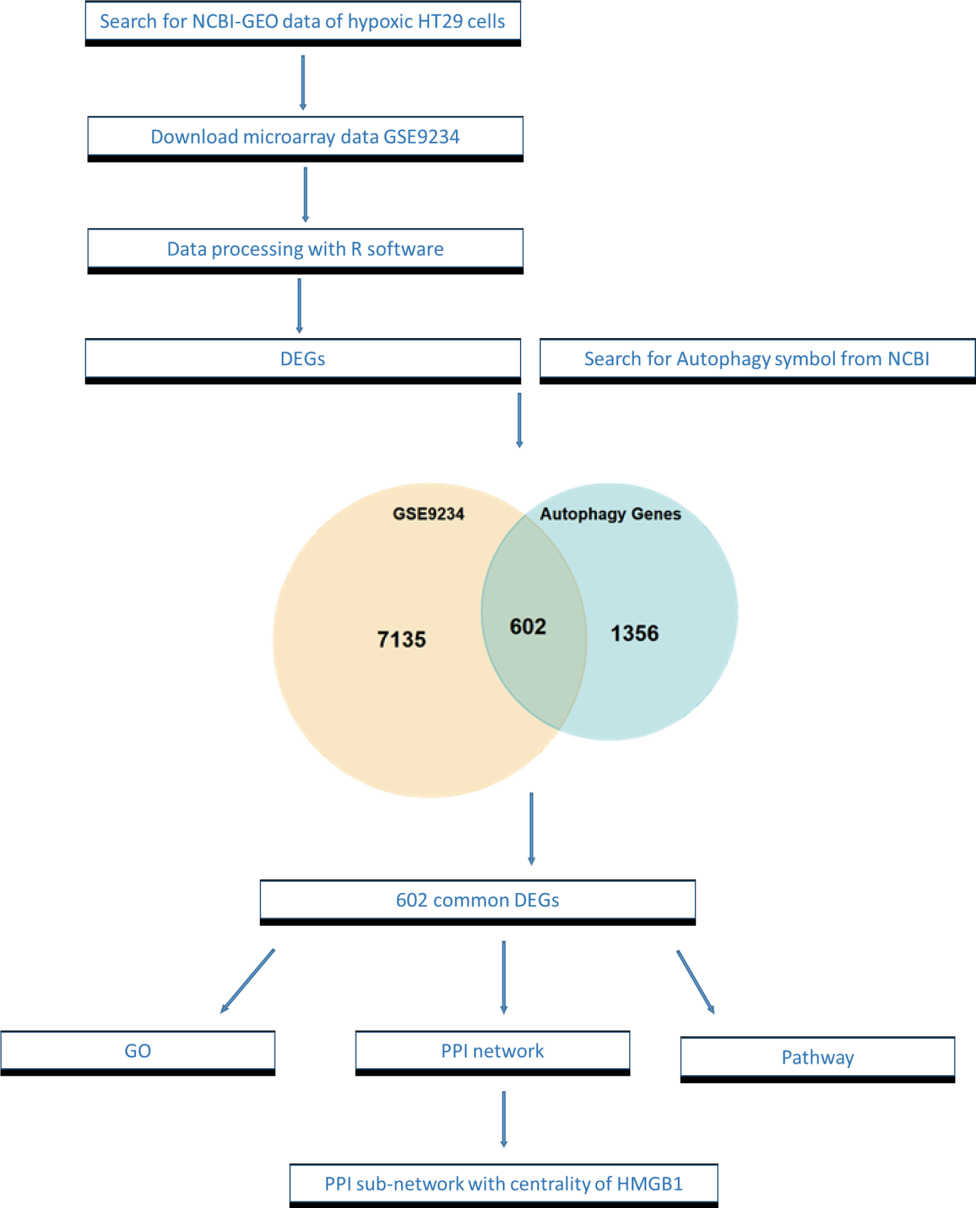
Overall diagram of data collection, processing, and analysis in this study. Identification of 602 autophagic DEGs from expression profile dataset (GSE9234) using R software. The cross area represents the commonly changed DEGs with retrieved autophagic symbols from NCBI. Statistically significant DEGs were defined by *p* < 0.05. GEO: Gene expression omnibus; DEGs: Differentially expressed genes; GO: Gene ontology; PPI: Protein–protein interaction

**Fig. 8:**
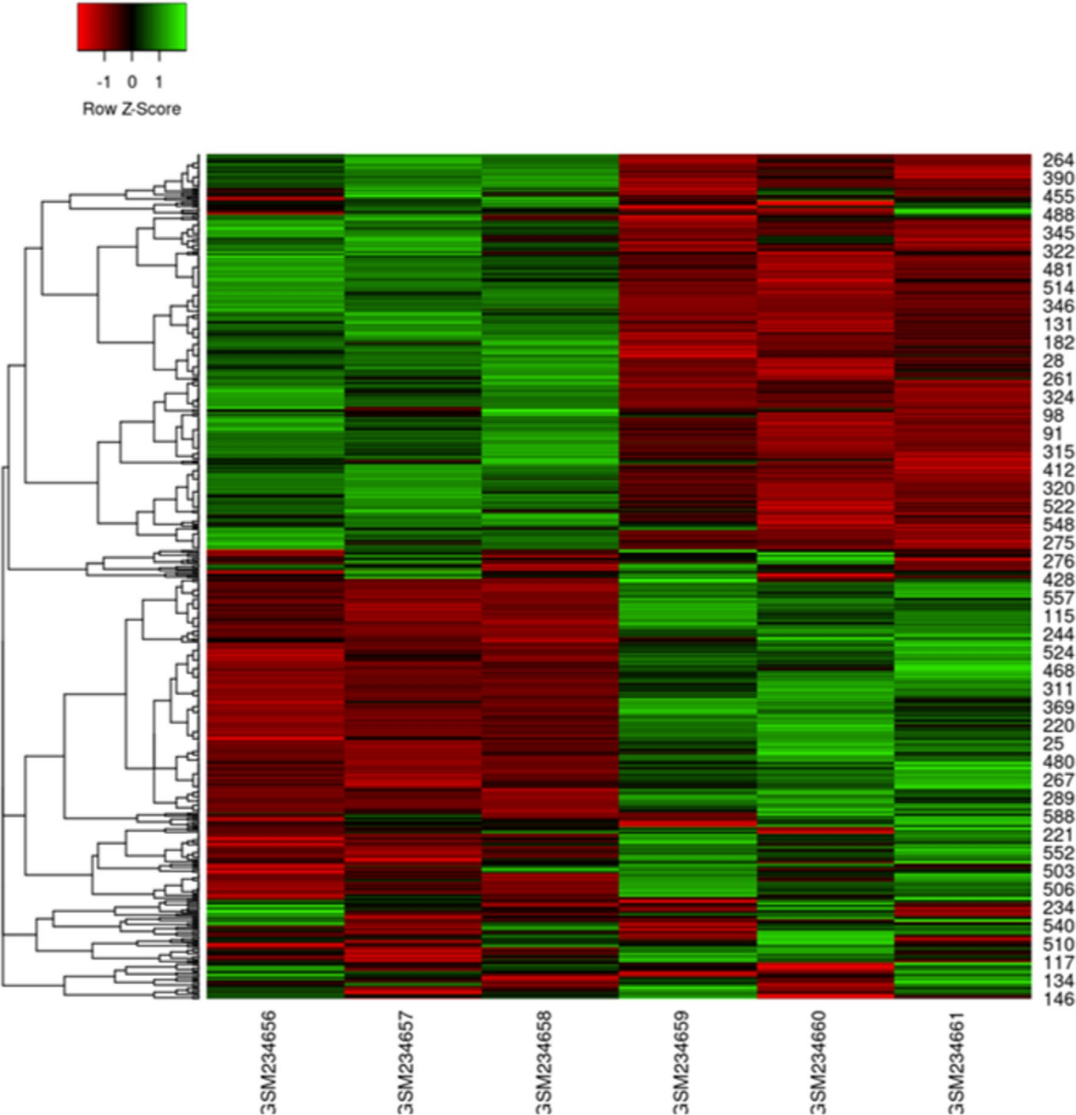
Heat map of of 602 autophagy-related genes were obtained from DEGs. Heatmap was plotted using heatmap.2 function of the R/Bioconductor package gplots. Hierarchical clustering of the DEGs was done by complete method with Euclidean distance. Red and green represent downregulation and upregulation, respectively. X-axis, samples; Y -axis, differentially expressed gene names

The overall design of this study is illustrated in Fig. 7. To gain some insight into how the hypoxia affects the expression of genes and to make a better understanding of HMGB1 interactions with other proteins, a publicly available mRNA expression profile dataset (GSE9234) was processed and normalized via R software. This dataset is consists of three samples of hypoxic conditioned HT29 cells and three samples of normoxic conditioned HT29 cells [25]. This dataset was the only microarray dataset available on hypoxic conditioned HT29 cell lines and could be helpful to predict the gene expression in infected HT29 cell lines during hypoxia or normoxia. The dataset contained 7135 differentially expressed genes (DEGs) (*P*-value < 0.01 and Fold change > 0.5), including 530 upregulated genes and 953 downregulated genes. Then, to identify the potential autophagy-related genes during hypoxia, 1356 autophagy symbol genes were retrieved from NCBI and compared to 7135 DEGs. A total of 602 autophagy-related genes were obtained from DEGs (Additional file 1: Table S1), of which 252 were upregulated, and 350 were downregulated (Fig. 8). ACSS2 and MYC were the most upregulated and downregulated differentially expressed genes, respectively.

### Protein–protein interaction (PPI) network analysis and pathway enrichment

**Fig. 9:**
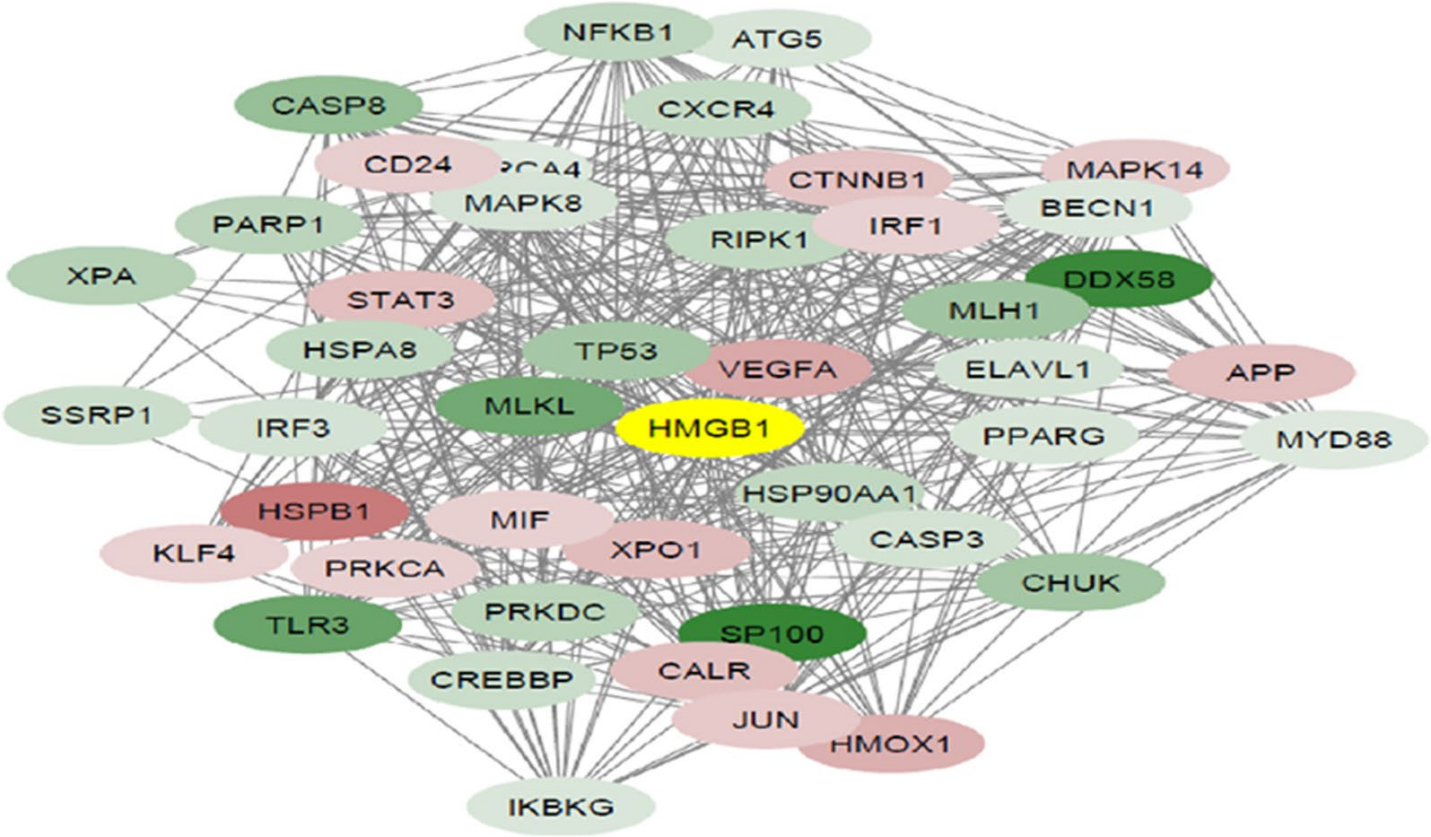
PPI-subnetwork of autophagic related genes during hypoxia with centrality of HMGB1. Red nods indicate upregulated genes, and green nods represent downregulated genes. The colour intensity in each node was proportional to fold change of expression in comparison to normoxic conditioned HT29 cell line. To visualize the nodes, Cytoscape spring-embedded layout algorithm was used

Using the STRING database, the PPI network of 602 autophagy-related DEGs was constructed and visualized in Cytoscape. The PPI network for a total of DEGs consisted of 556 nodes and 7523 edges. Then a sub-network that shows all the proteins interacting with HMGB1 was created. The sub-network with the centrality of HMGB1 protein was composed of 44 nodes and 421 edges, including 28 downregulated and 15 upregulated genes (Fig. 9). Gene ontology (GO) analysis was performed using DAVID. GO covers the three categories, including cellular component (CC), biological process (BP), and molecular function (MF). Upregulated DEGs were significantly enriched in response to stress (ontology: BP), nucleoplasm (ontology: CC), and enzyme binding (ontology: MF) (Additional file 1: Table S2). Downregulated DEGs were enriched in different GO terms, including positive regulation of multicellular organismal process (ontology: BP), nuclear euchromatin (ontology: CC), and enzyme binding (ontology: MF) (Table 1). Moreover, based on KEGG pathway analysis (Table 3), 89 and 71 pathways were significantly enriched in upregulated and downregulated DEGs, respectively.

**Table 3:** The KEGG pathway analysis of the top 10 differentially expressed genes associated with autophagy

Term	Pathway	Count	FDR
**Up-regulated DEGs**			
hsa05168	Herpes simplex infection	12	2.11E−15
hsa05161	Hepatitis B	11	5.91E−15
hsa04622	RIG-I-like receptor signaling pathway	9	5.14E−14
hsa05167	Kaposi's sarcoma-associated herpesvirus infection	11	5.14E−14
hsa05169	Epstein-Barr virus infection	10	3.61E−12
**Down-regulated DEGs**			
hsa04933	AGE-RAGE signaling pathway in diabetic complications	5	3.14E−06
hsa04010	MAPK signaling pathway	6	6.82E−06
hsa05418	Fluid shear stress and atherosclerosis	5	6.82E−06
hsa04370	VEGF signaling pathway	4	8.19E−06
hsa05164	Influenza A	5	8.42E−06

KEGG, Kyoto Encyclopaedia of Genes and Genomes

### Validation of differentially expressed genes by quantitative RT-PCR (qRT-PCR) analysis

**Fig. 10:**
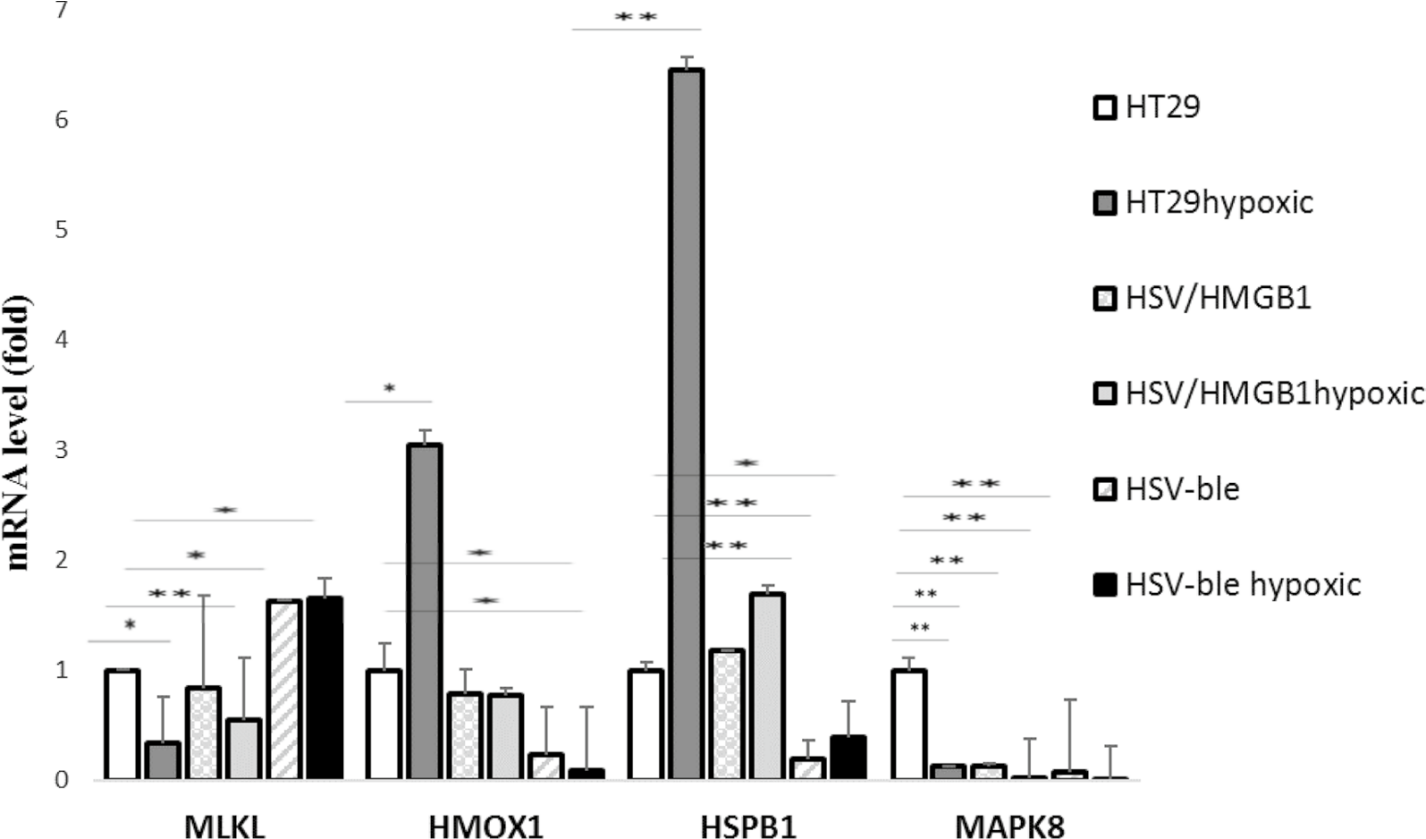
mRNA expression of MLKL, HSPB1, MAPK8, and HMOX1 in HT29cells infected with HSV-HMGB1 and HSV-ble under hypoxic and normoxic condition were analysed by reverse transcription-quantitative PCR. Gene expression levels were calculated based on the Delta-Delta Ct relative quantification. Three biological replicates were performed. *p < 0.05, and **p < 0.01

Based on GO analysis, we intended to focus on genes regulating autophagy in the PPI sub-network, including HMOX1, HSPB1, MLKL, and MAPK8. Then qRT-PCR was performed to determine whether the mRNA expression levels of these genes in HT29 cells infected with HSV-HMGB1and HSV-ble under hypoxic and normoxic conditions have been changed (Fig. 10). The results revealed that all four genes were differentially expressed under hypoxic conditions and were consistent with microarray dataset results. HSPB1 mRNA levels upregulated in HT29 cells infected with HSV-HMGB1 when subjected to hypoxia, but expression levels of HSPB1 in hypoxic and normoxic conditioned HT29 cells infected with HSV-ble was markedly decreased compared to the values in control cells (normoxic non-infected HT29 cells). Likewise, HMOX1 expression was downregulated in HT29 cells infected with HSV-ble under hypoxic or normoxic conditions, yet its expression remained unchanged in HSV-HMGB1 infected HT29 cells. MAPK8 expression values were significantly decreased in HT29 cells infected with either of the two viruses compared to untreated control HT29 cells. Interestingly, expression levels of MLKL increased in HT29 cells infected with HSV-ble during normoxia or hypoxia; by contrast, it was downregulated in HT29 cells infected with HSV-HMGB1 in response to hypoxia. As can be seen in Fig. 10, HSV-ble has caused a significant decrease in the transcription level of autophagy-related genes that are upregulated in hypoxia which might be correlated with the results of the XTT assay (Fig. 3) in which HSV-ble reduces the viability of HT29 cell during hypoxia.

## Discussion

In this study, we constructed an oHSV expressing HMGB1 which was reported as a potential anticancer protein capable of killing tumor cells during hypoxia or normoxia [10, 11]. Based on our results, HSV-HMGB1 recombinant virus exhibited higher cytotoxicity in HCT116 and SW480 cells under hypoxic and normoxic conditions than its parental virus; HSV- HMGB1 at MOI 0.1 in HT29 cells. However, showed lower cytotoxic activity compared with HSV-ble during hypoxia. He et al. reported that HMGB1 could promote tumor cell proliferation [26] which is consistent with our results (Fig. 3, HT29 hypoxic). Since HMGB1 could regulate apoptosis and autophagy, first, we examined the role of HMGB1 in apoptosis induction. Interestingly, the increase in the late apoptosis of HSV-HMGB1 infected HCT116 is distinct from the two other cell lines. The observed results might be the impact of P53 status of cancer cell lines; HCT116 cells express wild type P53. Livesey et al. explained that HMGB1 and P53 could form a complex by which cytoplasmic HMGB1 decreases and results in autophagy reduction. This data is in agreement with our confocal microscopy results where both P53 and HMGB1 reside in the nucleus in HCT116 cells infected with HSV-HMGB1 during normoxia or hypoxia (Fig. 6A, B). Then we examined the role of HMGB1 in autophagy induction. Evidence suggests that ICP 34.5 has a Beclin1 binding domain, which is essential to preclude autophagy. Therefore, the ICP 34.5 null mutants are expected to induce autophagy [27]. Likewise, US11, a true late gene product, could inhibit autophagy through interaction with PKR [21]. Therefore, to investigate whether autophagy plays a part in reducing the HSV-HMGB1 cytotoxicity in hypoxic conditioned HT29 cells, we examined the level of LC3-II expression in HCT116, SW480, and HT29 after infection with HSV- HMGB1 and HSV-ble during hypoxia and normoxia. Although LC3-II expression was observed in all the three hypoxic conditioned cell lines, among infected cells, LC3-II expression was only detectable in HT29 cells infected with HSV-HMGB1 under hypoxic conditions. Interestingly, Beclin1 expression was reduced in infected SW480 and HCT116 cells during hypoxia or normoxia. Still, the expression level of Beclin1 was relatively similar in HT29 cells infected with either of the two viruses under hypoxic or normoxic conditions. In the case of apoptosis and autophagy crosstalk, Wirawan et al. reported two caspase cleavage sites in Beclin1. They suggested that cleavage of Beclin1 by caspase not only disrupts the autophagic pathway, but Beclin1 fragments could induce apoptosis by translocation to the mitochondria, which could explain the downregulation of Beclin1 observed in western blot analysis in the infected SW480 and HCT116 cells (Fig. 5A) [28]. Of note, tumor cell lines analyzed in this study have different p53 status; as we mentioned earlier, HCT116 cells produce wild-type p53, whereas HT29 and SW480 cells contain mutant p53 [24]. Several shreds of evidence show that p53/HMGB1 complexes could regulate the cell fate; in the absence of p53, HMGB1 could translocate to the cytoplasm and form a complex with Beclin1, which increases the level of autophagy. On the contrary, cytosolic p53 might lead to autophagy reduction [23, 29, 30]. Confocal microscopy images demonstrated (Fig. 6A and B) that during hypoxia in HT29 cells infected with HSV-HMGB1, HMGB1 translocated to the cytoplasm as opposed to HCT116 cells in which HMGB1 was localized in the nucleus. These results support the idea that cytoplasmic translocation of HMGB1 promotes autophagy, well defined in previous studies [23]. Moreover, a recent study showed that cytoplasmic HMGB1 expression had been associated with high-grade CRC and poor prognosis. But, there was a better survival prognosis in CRC patients with nuclear HMGB1 expression in comparison with other patients [13]. We wondered why HT29 cells infected with HSV-HMGB1 showed a different pattern of HMGB1 localization in normoxic condition. Accumulated data have indicated that reactive oxygen species (ROS) decreases during hypoxia [31]. Livesey et al. explained that excessive ROS level enhances the interaction between HMGB1 and p53 by which cells maintain a balance between autophagy and apoptosis, which is in agreement with our confocal analysis where both HMGB1 and P53 reside in the nucleus of HT29 cells infected with HSV-HMGB1 during normoxia [23]. Additionally, Rochette et al. demonstrated that mutated p53 in SW480 cells is not truly non-functional, and some of the p53 functions could be restored [32]; based on this observation, there might be a mechanism by which mutated p53 retained its function to suppress autophagy [33].

Moreover, other groups have reported that HMGB1 could increase cell cytotoxicity. In the context of OV infection, HMGB1 could be bound to viral nucleic acids and serve as an adjuvant which ultimately promotes cell death [34, 35].

In the current study, we observed that HSV-HMGB1 induced autophagy in HT29 cells during hypoxia; therefore, to understand how this might arise at a molecular level, we first investigated the expression of genes involved in autophagy and determined their interaction with HMGB1 protein. Then, the PPI subnetwork was generated by using the DEGs involved in autophagy with the centrality of HMGB1. Further, GO analysis was performed to better illustrate how autophagy-related genes influence the different biological processes. Our results indicated that the highest score belonged to GO:0006950 involved in response to stress. Then, we identified key DEGs regulating autophagy (HMOX1, HSPB1, CASP3, BECN1, MAPK8, MLKL, and IKBKG). Finally, RT-qPCR was employed to explore whether overexpression of HMGB1 by HSV-HMGB1 could alter the expression of HSPB1, HMOX1, MAPK8, and MLKL genes which were selected based on topological parameters, i.e. degree in hypoxic conditioned HT29 cells. Following stress or unfavorable environment, the level of HSPB1 expression increases [36], and increased HSPB1 causes autophagy and inhibits apoptosis [37]. We showed that HSPB1 expression increased in HT29 cells infected with HSV-HMGB1 during hypoxia. Other investigators have found that over-expression of HMGB1 could also increase the level of HSPB1 protein [38], yet HSV1 would be able to suppress some anti-apoptotic genes and negative regulators, including HSPB1 [39].

Previous studies have shown that HMOX1 (HO-1) is strongly induced by hypoxia which is regulated by NRF2 [40]. A hypoxic environment enhances the nucleus translocation of NRF2, where it stimulates the expression of HO-1 and the increased expression of HO-1could fight against oxidative injury [41]. Besides, HO-1 is a negative regulator of HMGB1 release, and it has been reported that HMGB1 inhibition enhances the expression of NRF2, and suppression of NRF2 or HO-1significantly increases oxidative stress [42]. Our results indicated that HO-1 expression in HT29 cells infected with HSV-HMGB1 remained unchanged during hypoxia or normoxia; however, HO-1 was reduced by HSV-ble infection. Studies have shown that ICP34.5 null HSV1 could not interact with KEAP1 a negative regulator of NFR2, hence NFR2 nuclear translocation could not occur and HO-1 expression could be suppressed after ICP34.5 null HSV1 infection [43].

Necroptosis is another form of programmed cell death that depends on the RIP1/RIP3/MLKL axis [44]. HSV ICP6 could activate RIP3/MLKL and induce TNF independent necroptosis [45]. We observed that MLKL expression was increased in HSV-ble infection during normoxia or hypoxia, but MLKL expression was reduced in HT29 cells infected with HSV-HMGB1 after exposure to hypoxia; similarly, MLKL expression was downregulated in HT29 cells by hypoxia which is in alignment with some prior research [46]. Therefore, the involvement of necroptosis in HSV-ble infection should be considered but needs to be further elucidated.

Our primary objective in this study was to design an oncolytic HSV expressing HMGB1 to kill colorectal cancer cell lines, in particular during hypoxia; however, HSV-HMGB1 failed to destroy HT29 cells at MOI 0.1 under hypoxic conditions. It seems that autophagy played a role in the survival of HT29 cells infected with HSV-HMGB1 during hypoxia. However, other forms of programmed cell death, including ferroptosis and necroptosis, will be addressed in our future study. Moreover, additional work is needed to explore the caspase activity and ROS production in infected cell lines during hypoxia and normoxia. In addition, the safety for the insertion of HMGB1 in the oncolytic virus in an animal models will be studied in our future study.

## Conclusion

Our results herein demonstrate that CRC cells respond to oHSV expressing HMGB1 differently, which is likely dependent on the profile of the tumor cell microenvironment and tumor cell type itself. Moreover, HMGB1 binding partners and posttranslational modifications may largely affect its cytotoxicity. Moreover, our data imply that to advance the oncolytic virus cancer treatment and comprehend why specific tumor cells respond to therapy, and others do not react, it is imperative to identify the cancer cells’ characteristics and intracellular signaling and especially comprehend its interaction with the modified oncolytic viruses.

## Material and method

### Cell culture

Three Vero, BHK, and HEK293T cell lines originated from the kidney of African green monkey, Baby hamster, and human embryo, respectively, and also three human colon cancer cells including one colorectal carcinoma (HCT116) and two colorectal adenocarcinomas (SW480 and HT-29) cell lines were purchased from the national cell bank of Iran (NCBI). Cells used in the experiments were cultured in RPMI 1640 (Gibco, New Zealand) or Dulbecco's modified Eagle's medium (DMEM) (Gibco, New Zealand) supplemented with 10% FBS (Gibco, New Zealand) and were incubated in a humidified incubator supplied with 5% CO_2_ at 37 °C.

### Cancer cell adaptation to hypoxia

SW480, HCT116, and HT29 have seeded in a T25 flask and cultured in DMEM and RPMI medium supplemented with 10% FBS. The cells were repeatedly incubated in the hypoxic conditions in Anoxomat chambers (Mart Microbiology, Lichtenvoorde, The Netherlands) (1% O_2_) for four h, and then incubated in a standard culture environment (5% CO_2_, 95% air) at 37 °C for 48–72 h. Cells were treated twice weekly. Hypoxic-conditioned cell lines were generated after 20 exposures to hypoxia [47].

### Generation of HMGB1 shuttle vectors

A 668-bp fragment of HMGB1-coding sequence (NM_002128.7) with *Bam*HI and *Xho*I restriction sites was synthesized. Afterward, the HMGB1 gene was sub-cloned into pcDNA3.1 + at *Bam*HI and *Xho*I sites. To generate a shuttle vector, the HMGB1expression cassette (CMV-HMGB1-BGH polyA signal) was digested with *Mlu*I and *Pvu*II and replaced with the GFP reporter gene in pSL-HomoF1TK- GFP -HomoF2TK (generated in our previous work) at *Mlu*I and *Eco*RV sites. This vector was used to insertion the HMGB1 coding sequence at the *tk* locus in the HSV-ICP34.5- Blecherry TK-GFP to generate new recombinant HSV-HMGB1. Construction of HSV-ICP34.5- Blecherry was described elsewhere [48].

### Generation of HSV-HMGB1

To generate HSV-HMGB1, the GFP coding sequence was replaced with the HMGB1 gene by homologous recombination using pSL-HomoF1TK-HMGB1-HomoF2TK plasmid and HSV-ICP34.5- Blecherry TK- GFP as a parental virus. The pSL-HomoF1TK-HMGB1-HomoF2TK plasmid was transfected using Lipofectamine 3000 (Invitrogen, USA) on a 90% monolayer of BHK21 cells grown in six-well plates. On the following day, transfected cells were infected with HSV-ICP34.5- Blecherry TK-GFP at MOI 1. The cells were incubated at 37 °C in 5% CO_2_ for 24–36 h until the observation of cytopathic effects. Next, recombinant viruses were isolated by three passages in a monolayer Vero cell, overlaid with DMEM containing 2% FBS and 1.5% methylcellulose. To verify the insertion of HMGB1, viral DNA was purified by the phenol/chloroform method for PCR analysis. PCR was performed with the.

hmgb1F 5′-CTTCTTAGGATCTCCTTTGC-3′, and tkF 5′- ACAGGTCGCCGTTGGGGGCCA-3′ primers (Additional file 1: Table S2). Thereafter, the isolated recombinant virus was titrated with plaque assay on Vero cells and stained with Giemsa for 20 min to visualize the plaques. Purified recombinant virus at 10^7^ PFU/ml concentration was stored at − 70 °C for further applications.

### Viral growth analysis

The ability of HSV-HMGB1 to replicate within HCT116, SW480, and HT29 cells was evaluated by viral growth analysis. 2 × 10^5^ cells per well were plated into 6-well plates. Cells were then infected with HSV-HMGB1 (MOI 0.1) and with HSV-ble (MOI 0.1). Cells and media were harvested at 10, 24, 48, and 72 h post-infection. After three cycles of freeze–thaw, a standard plaque assay was performed on Vero cells to evaluate viral titers. All readings were performed in duplicate.

### Immunoblot analysis

HSV-HMGB1 and HSV-ble infected HCT116, SW480 and HT29 cells and uninfected SW480, HCT116 and HT29 cells under hypoxic and normoxic conditions were lysed with RIPA buffer. Lysates were centrifuged at 15,000 ×*g* at 4 °C for 10 min, and the supernatants were collected. Protein concentrations were quantified using the BCA method (Sigma-Aldrich, United States). 50 µg of sample proteins were separated on 10–12% SDS polyacrylamide gel and transformed into polyvinylidene fluoride (PVDF) membrane. The membrane was then incubated with primary antibody and peroxidase-conjugated secondary antibody. Antibody binding was visualized by ECL Western Blotting Substrate (Thermo Scientific™ Pierce™, United States) according to the manufacturer’s instruction. The antibodies utilized were rabbit polyclonal to HMGB1(ab18256; Abcam, UK) diluted at a ratio of 1:1000, rabbit polyclonal to LC3 (ab51520**;** Abcam, UK) diluted at a ratio of 1:3000, rabbit polyclonal to Beclin1 diluted at a ratio of 1:2000 (ab92389**;** Abcam, UK), mouse monoclonal antibody to β-actin (A2228; Sigma-Aldrich, United States), anti-mouse IgG and anti-rabbit IgG peroxidase-conjugated secondary antibody (A9309, A9169; Sigma-Aldrich, United States).

### 2,3-bis-(2-methoxy-4-nitro-5-sulfophenyl)-2H-tetrazolium-5-carboxanilide) assay

**Fig. 11:**
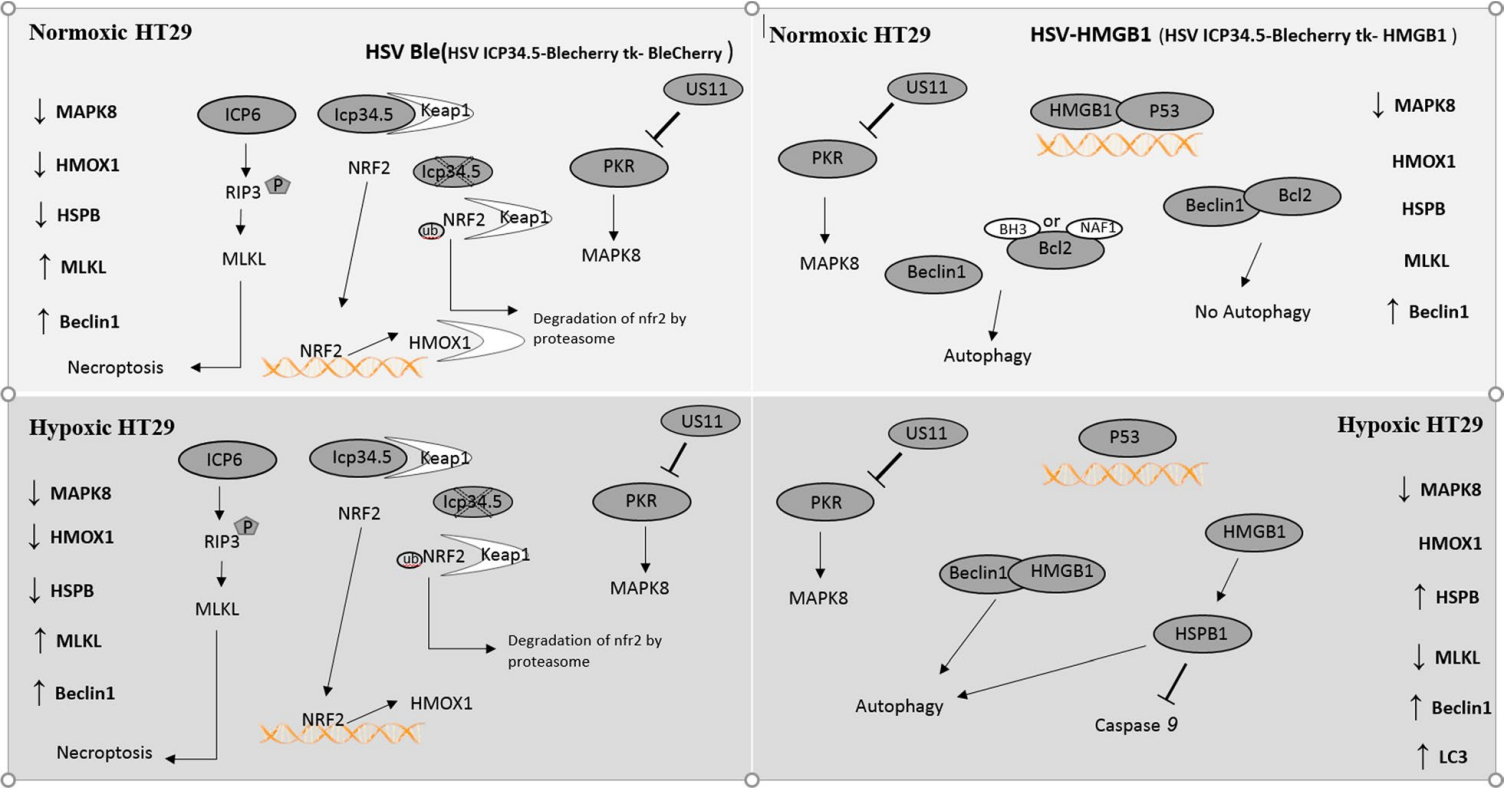
A hypothetical schematic illustration of pathways in infected HT29 cells (HSV-ble and HSV-HMGB1) during hypoxia and normoxia. Changes in the expression of MLKL, HSPB1, MAPK8, and HMOX1 genes are shown in each panel

HSV-HMGB1 and HSV-ble Infected HCT116, SW480, and HT29 cells and uninfected HCT116, SW480, and HT29 cells were plated at a density of 10^4^ cells per well in 96-well plates under hypoxic and normoxic conditions. The plated cells were incubated overnight in 5% CO_2_ at 37 °C. For the hypoxic survival experiments, the cells were infected with HSV-HMGB1 and HSV-ble (MOI 10, 1, 0.1, and 0.01) and placed in the anaerobic chamber for 72 h. For the normoxic survival experiments, the cells were infected with HSV-HMGB1 and HSV-ble (MOI 10, 1, 0.1, and 0.01) and placed in a standard culture environment (5% CO_2_, 95% air) at 37 °C for 72 h. Then cytotoxicity was determined using the XTT assay (Roche, Switzerland). Following 72 h incubation, XTT reagent was added per well. After 4 h at 37 °C, absorbance at 450 nm was determined using a microplate reader (El×800, BioTek Instruments Inc.). The reported values were the result of triplicate determinations (see Fig. 11).

### Annexin V/propidium iodide (PI) staining assay

Annexing V-Alexa fluor 488 apoptosis detection kit (Roche, Switzerland) was used to assess the apoptosis according to the manufacturer's protocol. HCT116, SW480, and HT29 cells were infected in 6-well plates with HSV-HMGB1 and HSV-ble at MOI 1 for 24 h under hypoxic and normoxic conditions. Cells were harvested and washed with cold phosphate-buffered saline (PBS) and then resuspended in 1 × binding buffer. Cells (1 × 10^6^) were incubated with Annexin V-Alexa fluor and PI for 15 min and analyzed by flow cytometry. Flow cytometry was performed with a Cyflow flow cytometer (Partec, Germany), and apoptosis was analyzed using Flowmax software.

### Confocal fluorescence microscopy

3 × 10^4^ HT29 and HCT116 Cells were plated on a coverslip and infected with HSV-HMGB1 at MOI 1 under hypoxic and normoxic conditions for 24 h. The plated cells were then fixed in 4% paraformaldehyde in PBS for 10 min and permeabilized with 0.2% Triton X-100 for 10 min. Cells were incubated with primary and secondary antibodies for one h sequentially. For nuclear counterstaining, DRAQ5 (reagent for DNA staining) was used at a 1:200 dilution ratio for 15 min and then mounted using Vectashield mounting medium (Vector Laboratories). Confocal laser-scanning microscope Leica TCS SP 8 (Leica Microsystems, Mannheim, Germany) was used to analyze the cells. Mean fluorescence intensity of HMGB1 and DRAQ5 in HT29 cells infected with HSV-HMGB1 and HCT116 cells infected with HSV-HMGB1 during normoxia and hypoxia were quantified using ImageJ software. The antibodies used were rabbit polyclonal against HMGB1(ab18256; Abcam, UK) diluted at a ratio of 1:200, mouse p53 monoclonal antibody DO-1 (AHO0152; Thermo, United States) diluted at a ratio of 1:100, FITC goat anti-mouse IgG antibody (F0257; Sigma-Aldrich, United States) and FITC goat anti-rabbit IgG antibody (F0382; Sigma-Aldrich, United States) diluted at 1:500 ratios.

### Microarray data and identification of differentially expressed genes (DEGs)

GSE9234, microarray expression profile dataset, submitted by Guimbellot JS et al. [25], was downloaded from the GEO database (http://www.ncbi.nlm.nih.gov/geo). The datasets contained three samples of hypoxic conditioned and three samples of normoxic conditioned HT29 cells. The database was built on the GPL570 platform on the Affymetrix Human Genome U133 Plus 2.0 Array. In this study, microarray data was first normalized and background-corrected. Then limma package (linear models for microarray data) [49] in R language was used to identify DEGs between hypoxic cells and oxygenated controls. Benjamin & Hochberg method was used to adjust *P*-values and were calculated separately for each DEGs [50]. FDR < 0.05 and log fold change (FC) > 0.5 were used as thresholds.

### Functional and pathway enrichment analyses

Gene Ontology database (GO; www.geneontology.org) (GO) analysis [51], and Kyoto encyclopedia of Genes and Genomes (KEGG) [52], pathway enrichment analyses, were performed using DAVID. Database for Annotation, Visualization and Integrated Discovery (DAVID) 6.8 (http://david.abcc.ncifcrf.gov/) is an online analysis platform that provides comprehensive biological and functional information associated with an extensive gene list [53]. *P* < 0.05 was considered to indicate a statistically significant difference.

### Construction of a PPI network and analysis

In this study, Search Tool for the Retrieval of Interacting Genes (STRING https://string-db.org/cgi), an online database that predicts protein–protein interactions [54], was used to construct a PPI network of identified DEGs. A confidence score of 0.4 was set as the cut‑off criterion. The protein interaction network was visualized using the Cytoscape software [55].

### RT‑qPCR analysis

Trizol reagent (TaKaRa, Kusatsu, Shiga, Japan) was used for RNA isolation of HSV-HMGB1 and HSV-ble infected HT29 cells and non-infected HT29 cells during hypoxia and normoxia. Then RNA samples were reversely transcribed to cDNA by QIAGEN Reverse Transcription Kit. Subsequently, cDNA quantification was performed by the qRT-PCR method using SYBR Green master mix (Amplicon). The reaction conditions were 95 °C for 10 min, 40 cycles of 95 °C for 10 s, 60 °C for 30 s, and 72 °C for 30 s. The 18S rRNA was used as an internal reference control. Gene expression levels were calculated based on the Delta-Delta Ct relative quantification (Additional file 1: Table S2).

### Statistical analysis

Statistical analyses were performed using the student’s *t*-test with GraphPad Prism 8 software (GraphPad Prism, San Diego, CA). The results were presented as the mean ± s.d. *P*-values < 0.05 were considered statistically significant.

## Supplementary Information

**Additional file 1:** **Figure S1.** Fluorescence intensities were measured using the ImageJ software. Bar graphs show mean fluorescence intensity (MFI) in uninfected and HSV-ble infected HT29 cells. There were no significant differences in HMGB1 expression between HSV-ble infected and non-infected HT29 cells. **Figure S2.** XTT assays with CRC cells under normoxic and hypoxic conditions demonstrating a dose-dependent cytotoxicity 72 hours after infection with HSV-HMGB1, and HSV-ble. The relative cell viability was normalised to that of the control (non- infected CRC cells at 72 hr). Three independent experiments were performed. data are shown as means ± sd. n=3, *p < 0.05. **p < 0.01, and ***p < 0.001. **Table S1.** Total of 602 autophagy DEGs obtained from GSE9234 dataset. **Table S2.** Gene Ontology analysis of the most significant upregulated and downregulated differentially expressed autophagy genes. **Figure S3.** Localization of HMGB1 in the nuclei was significantly higher in HT29 cell lines infected with HSV-HMGB1 during normoxia than in HT29 cell lines infected with HSV-HMGB1 during hypoxia. To check the localization of the desired protein, nuclei were selected based on draq5 staining and then intensity of green dye was measured in the same area, and divided to draq5 intensity. all measurements were normalized to the background intensity of cell free area of the same picture. Fluorescence intensities were measured using the ImageJ software. Bar graphs show mean fluorescence intensity of HMGB1 (green) and DRAQ5 (blue) in HT29 and HCT116 cell lines infected with HSV-HMGB1 during normoxia than in HT29 and HCT116 cell lines infected with HSV-HMGB1 during hypoxia. ***p < 0.001

## Data Availability

The data that support the findings of this study are available in (GEO) at (https://www.ncbi.nlm.nih.gov/geo/).

## Abbreviations

HMGB1: High mobility group box 1
CRC: Colorectal cancer cell
HSV-1: Herpes simplex virus type 1
oHSV-1: Oncolytic HSV type 1
NLS: Nuclear localization signals
LC3: Microtubule-associated protein light chain 3
DEGs: Differentially expressed genes
GO: Gene ontology
CC: Cellular component
BP: Biological process
MF: Molecular function
DAVID: Database for Annotation, Visualization and Integrated Discovery
KEGG: Kyoto encyclopedia of Genes and Genomes
PPI: Protein–protein interaction
qRT-PCR: Real-time quantitative PCR
