# Supplementary material for: Cell type-specific response of colon cancer tumor cell lines to oncolytic HSV-1 virotherapy in hypoxia
Source: Cancer Cell Int. 2022 Apr 27;22:164. doi: 10.1186/s12935-022-02564-4 (PMC9044800; doi:10.1186/s12935-022-02564-4)
Supplement: Supplementary file 1 — Additional file 1: Figure S1. Fluorescence intensities were measured using the ImageJ software. Bar graphs show mean fluorescence intensity (MFI) in uninfected and HSV-ble infected HT29 cells. There were no significant differences in HMGB1 expression between HSV-ble infected and non-infected HT29 cells. Figure S2. XTT assays with CRC cells under normoxic and hypoxic conditions demonstrating a dose-dependent cytotoxicity 72 hours after infection with HSV-HMGB1, and HSV-ble. The relative cell viability was normalised to that of the control (non- infected CRC cells at 72 hr). Three independent experiments were performed. data are shown as means ± sd. n=3, *p < 0.05. **p < 0.01, and ***p < 0.001. Table S1. Total of 602 autophagy DEGs obtained from GSE9234 dataset. Table S2. Gene Ontology analysis of the most significant upregulated and downregulated differentially expressed autophagy genes. Figure S3. Localization of HMGB1 in the nuclei was significantly higher in HT29 cell lines infected with HSV-HMGB1 during normoxia than in HT29 cell lines infected with HSV-HMGB1 during hypoxia. To check the localization of the desired protein, nuclei were selected based on draq5 staining and then intensity of green dye was measured in the same area, and divided to draq5 intensity. all measurements were normalized to the background intensity of cell free area of the same picture. Fluorescence intensities were measured using the ImageJ software. Bar graphs show mean fluorescence intensity of HMGB1 (green) and DRAQ5 (blue) in HT29 and HCT116 cell lines infected with HSV-HMGB1 during normoxia than in HT29 and HCT116 cell lines infected with HSV-HMGB1 during hypoxia. ***p < 0.001 [file 12935_2022_2564_MOESM1_ESM.docx]

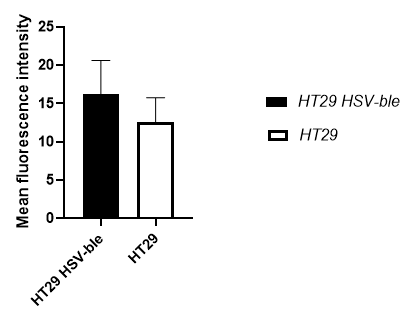

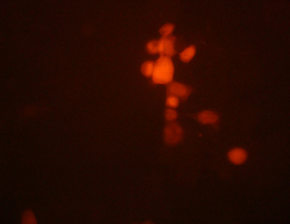

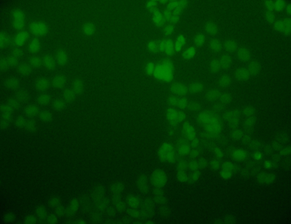


Fig S1. Fluorescence intensities were measured using the ImageJ software. Bar graphs show mean fluorescence intensity (MFI) in uninfected and HSV-ble infected HT29 cells. There were no significant differences in HMGB1 expression between HSV-ble infected and non-infected HT29 cells.


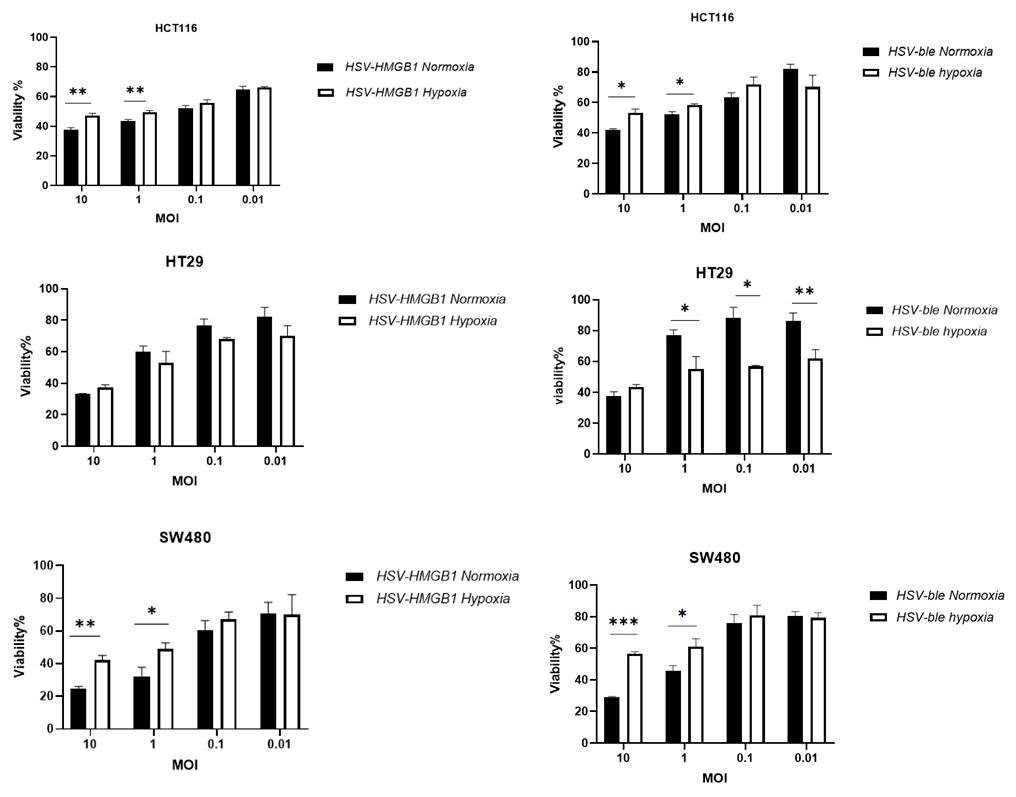


Fig S2. XTT assays with CRC cells under normoxic and hypoxic conditions demonstrating a dose-dependent cytotoxicity 72 hours after infection with HSV-HMGB1, and HSV-ble. The relative cell viability was normalised to that of the control (non- infected CRC cells at 72 hr). Three independent experiments were performed. data are shown as means ± sd. n=3, *p < 0.05. **p < 0.01, and ***p < 0.001.

Table S1. Total of 602 autophagy DEGs obtained from GSE9234 dataset.

Table S2. Gene Ontology analysis of the most significant upregulated and downregulated differentially expressed autophagy genes.


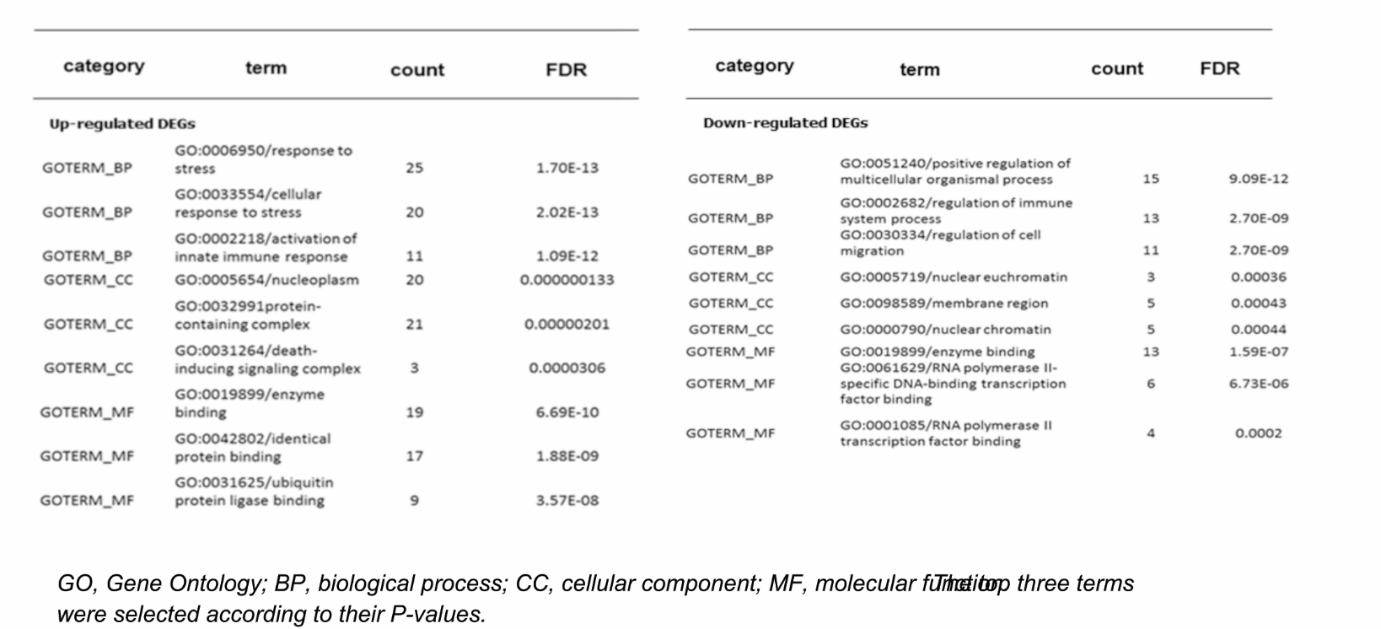


Table S2. Table of RT-PCR primer sequences


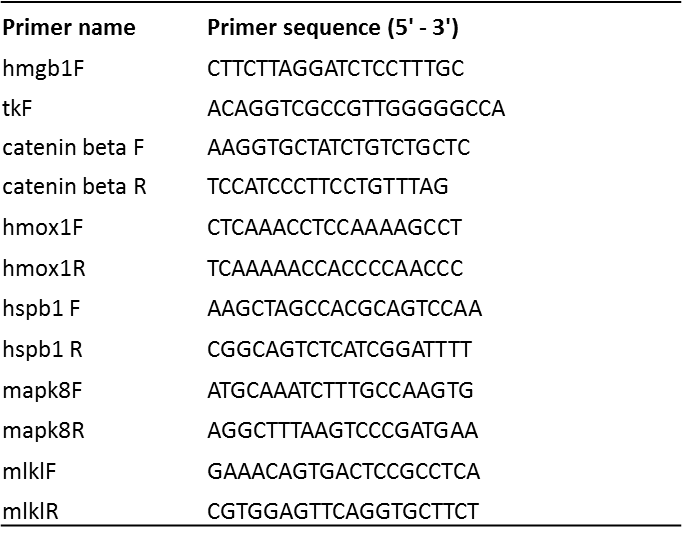


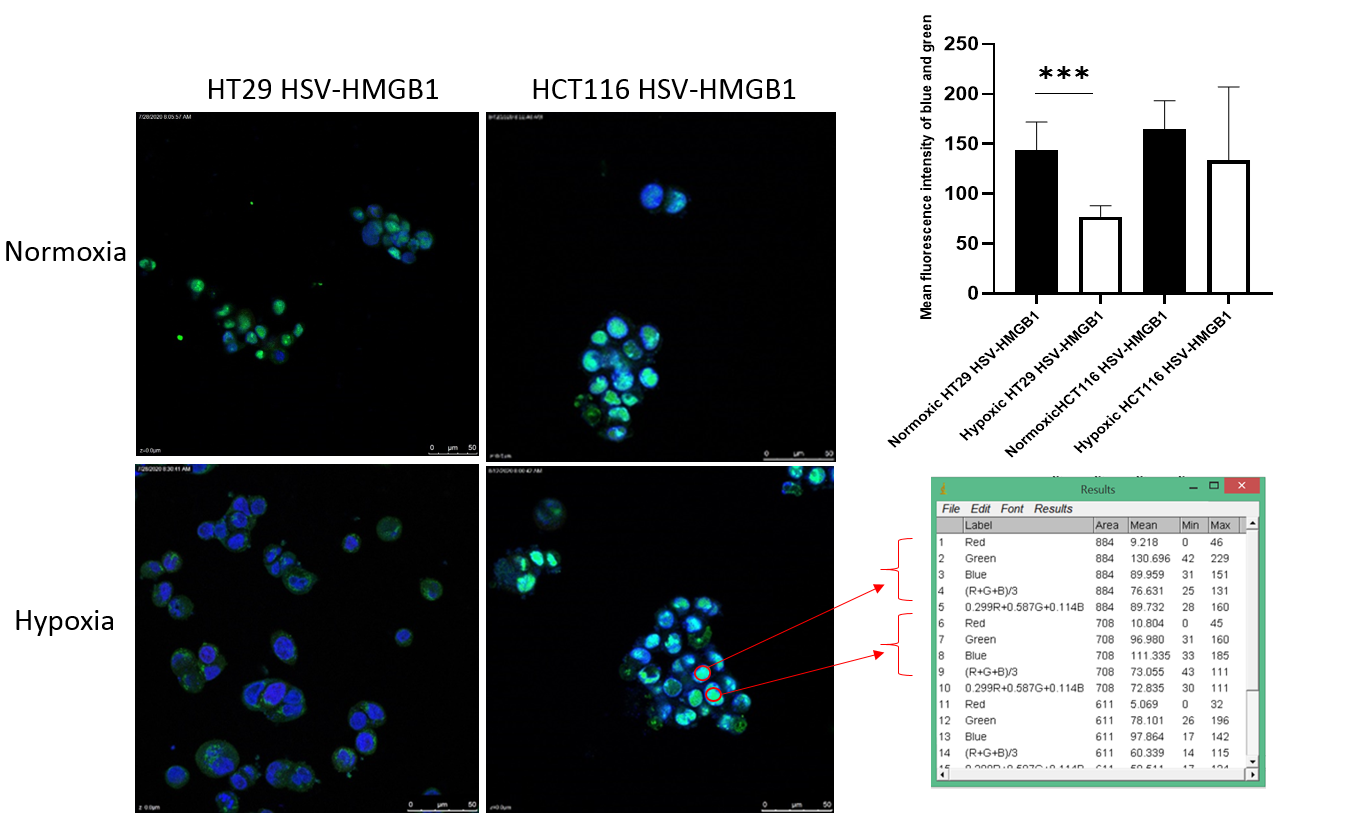


Fig S3. Localization of HMGB1 in the nuclei was significantly higher in HT29 cell lines infected with HSV-HMGB1 during normoxia than in HT29 cell lines infected with HSV-HMGB1 during hypoxia. To check the localization of the desired protein, nuclei were selected based on draq5 staining and then intensity of green dye was measured in the same area, and divided to draq5 intensity. all measurements were normalized to the background intensity of cell free area of the same picture. Fluorescence intensities were measured using the ImageJ software. Bar graphs show mean fluorescence intensity of HMGB1 (green) and DRAQ5 (blue) in HT29 and HCT116 cell lines infected with HSV-HMGB1 during normoxia than in HT29 and HCT116 cell lines infected with HSV-HMGB1 during hypoxia. ****p* < 0.001
